# Animal models with group-specific additive genetic variances: extending genetic group models

**DOI:** 10.1186/s12711-019-0449-7

**Published:** 2019-02-28

**Authors:** Stefanie Muff, Alina K. Niskanen, Dilan Saatoglu, Lukas F. Keller, Henrik Jensen

**Affiliations:** 1 Institute of Evolutionary Biology and Environmental Studies, University of Zurich, Winterthurerstrasse 190, Zurich, Switzerland; 2 Department of Biostatistics, Epidemiology, Biostatistics and Prevention Institute, University of Zurich, Hirschengraben 84, Zurich, Switzerland; 3 Department of Biology, Centre for Biodiversity Dynamics, Norwegian University of Science and Technology, Høgskoleringen 5, Trondheim, Norway; 4 Department of Ecology and Genetics, University of Oulu, P.O. Box 3000, Oulu, Finland; 5 Zoological Museum, University of Zurich, Karl-Schmid-Strasse 4, Zurich, Switzerland

## Abstract

**Background:**

The *animal model* is a key tool in quantitative genetics and has been used extensively to estimate fundamental parameters, such as additive genetic variance or heritability. An implicit assumption of animal models is that all founder individuals derive from a single population. This assumption is commonly violated, for instance in crossbred livestock or when a meta-population is split into genetically differentiated subpopulations. Ignoring that base populations are genetically heterogeneous and thus split into different ‘genetic groups’ may lead to biased parameter estimates, especially for additive genetic variance. To avoid such biases, genetic group animal models, which account for the presence of more than one genetic group, have been proposed. Unfortunately, the method to date is only computationally feasible when the breeding values of the groups are allowed to differ in their means, but not in their variances.

**Results:**

We present an extension of the animal model that permits estimation of group-specific additive genetic variances. This is achieved by employing group-specific relatedness matrices for the breeding value components to different genetic groups. We derive these matrices by decomposing the full relatedness matrix via the generalized Cholesky decomposition, and by scaling the respective matrix components for each group. We propose a computationally convenient approximation for the matrix component that encodes for the Mendelian sampling variance, and show that this approximation is not critical. In addition, we explain why segregation variances are often negligible when analyzing the complex polygenic traits that are frequently the focus of evolutionary ecologists and animal breeders. Simulations and an example from an insular meta-population of house sparrows in Norway with three distinct genetic groups illustrate that the method is successful in estimating group-specific additive genetic variances, and that segregation variances are indeed negligible in the empirical example.

**Conclusions:**

Quantifying differences in additive genetic variance within and among populations is of major biological interest in ecology, evolution, and animal and plant breeding. The proposed method allows to estimate such differences for subpopulations that form a connected set of populations, and may thus also be useful to study temporal or spatial variation of additive genetic variances.

**Electronic supplementary material:**

The online version of this article (10.1186/s12711-019-0449-7) contains supplementary material, which is available to authorized users

## Background

Quantifying the (causal) relationships between genes and observed phenotypic traits is a central task of empirical studies of adaptive evolution [1, 2] and of plant and animal breeding [3]. The *animal model* [4–6] has become a popular statistical approach to disentangle genetic effects on a phenotype from other factors that may induce phenotypic similarities among relatives, such as shared environmental effects [7], inbreeding [8], or individual traits such as age or sex [9, 10]. Fundamental to the animal model is information on how animals are related to each other, information typically obtained from pedigree data [11, 12], from genomic data (e.g.[13, 14]), or a combination of both [15]. Pedigrees are still the most commonly used source of relatedness information in animal models (e.g. [16, 17]), in part because the use of pedigrees leads to models that are computationally efficient.

All pedigrees necessarily start with a founder generation of individuals with unknown parents, so-called ‘phantom parents’ [17]. The animal model assumes that all founder individuals stem from a single, genetically homogeneous baseline population, and that the model estimates additive genetic variance (denoted as $\sigma _A^2$) of the respective base population. When the homogeneity assumption is violated, for example in the presence of immigrants from another population or in crossbred livestock breeds, estimates of $\sigma _A^2$ may be biased [18, 19]. To address this problem, animal breeders developed animal models with genetic groups, briefly denoted as *genetic group models* (e.g. [20]), and these are now also receiving attention in evolutionary ecology [17, 21, 22]. The main idea behind genetic group models is that accounting for differences in mean breeding values may reduce or eliminate the bias [17]. However, current genetic group models have an important key limitation: genetic groups are allowed to differ in mean breeding value, but are assumed to have the same additive genetic variance. This homogeneity assumption is violated in some animal breeding applications [23–25], and is likely also violated in many natural populations, where source populations of immigrants may differ in additive genetic variance, for example due to differences in effective population size (genetic drift) and selection regimes (e.g. [26–28]). In fact, different populations or ecotypes within the same species have been found to differ in their additive genetic (co)variances in plants (e.g. [29]), invertebrates (e.g. [30]), and vertebrates (e.g. [31]). Because additive genetic variances determine the evolutionary potential of phenotypic traits [1, 32], and because of the fundamental importance of understanding the processes that shape additive genetic variances, as well as the consequences that selection will have on the rate and direction of evolution within and across populations, it is essential to be able to estimate the additive genetic variance of each baseline population in the presence of interbreeding genetic groups.

Aiming for better predictions of breeding values in crossbred populations, animal breeders have suggested approaches that account for heterogeneous additive genetic variances across genetic groups [19, 24, 33]. One drawback of these models is that they rapidly become unfeasibly complex, because variability of the genetic values must now be split into components from the pure breeds *plus* components due to segregation terms when breeds are mixed, which requires that the respective segregation variance terms enter the model. Segregation variance refers to the increase in variance caused by differences in allele combinations, average allelic effects, and linkage disequilibrium at and between loci underlying the phenotype in the mixing breeds, as in $F_1$ and $F_2$ generations of line crosses [34–36]. The respective terms can be large in crossbreeding applications [1, p. 11]. However, as we explain in detail below, we expect the segregation variance from crossing different genetic groups in wild study populations to be small for many traits of interest, so that omitting it from the animal model does not lead to significant bias.

Thus, here we use the simplified models without segregation terms to derive genetic group models that allow for group-specific additive genetic variances. In order to properly consider each individual’s genetic contribution to the actual population, we additively split the breeding value of each individual into group-specific components, similar to the approach suggested by García-Cortes and Toro [33]. For each group, the components that stem from the same genetic group covary according to a group-specific relatedness matrix. The main challenge is to find these matrices. Instead of implementing a recursive procedure to calculate the inverse of the additive genetic covariance matrix [23, 33], we propose to derive group-specific relatedness matrices by first decomposing the full relatedness matrix (disregarding genetic groups) via a generalized Cholesky decomposition (as described by [12]), and then appropriately scaling the respective matrix components for each group. This procedure has the advantage that we can use the same mathematical approach that Henderson and Quaas developed to decompose a single population’s relationship matrix [12, 37]. Moreover, by incorporating multiple inverse relatedness matrices into a single mixed model, existing algorithms for the analysis of single populations can easily be extended to genetic group animal models with group-specific additive genetic variances.

In the following, we first summarize the current state of genetic group models and then give a detailed description of the extension to heterogeneous group-specific additive genetic variances. We illustrate the performance of our method with a simulation study and an application to a meta-population of house sparrows (*Passer domesticus*) in Norway, where genetic groups are determined by geographical properties of the bird’s natal island population. By also fitting a model that includes a segregation term to the sparrow data, we illustrate that omitting segregation variances is unproblematic in such applications. We also provide a short tutorial including $\mathsf {R}$ code for the analysis, and discuss opportunities and limitations of our extended genetic group model.

## Methods

### The animal model for genetic groups with homogeneous variances

The basic animal model for a phenotypic measurement $y_i$ of an individual *i* ($1\le i \le n$) is

1 $$\begin{aligned} y_i = \mu + a_i + e_i \ , \end{aligned}$$

with population mean $\mu$, environmental component $e_i \sim \mathsf {N}(0,\sigma _E^2)$ with environmental variance $\sigma _E^2$, and breeding values distributed as ${\mathbf {a}}^\top = (a_1, \ldots , a_n)^\top \sim \mathsf {N}({\mathbf {0}},\sigma _A^2 {\mathbf {A}})$ with additive genetic variance $\sigma _A^2$ and additive genetic relatedness matrix ${\mathbf {A}}$ that represents the relatedness among individuals [5, 12]. Model (1) is often extended by fixed effects (such as sex or age) and by additional random effects that account for permanent environmental conditions (see e.g. [6]). In all cases, the underlying assumption is that all animals in the analysis derive from the same genetic population, and that the breeding values ($a_i$) encode for the deviation from the mean of this population and thus have a mean of zero.

As noted by animal breeders a long time ago, these assumptions are frequently violated, for example in crossbred populations from genetically differentiated breeds. In such cases it is necessary to allow for differences in mean breeding values among animals with different genetic origins [18, 20], as otherwise the prediction of breeding values or the estimates of the base populations’ additive genetic variances are biased. Let us denote by a founder population a set of animals with unknown true parents, whose phantom parents comprise the base population, and assume that *r* base populations exist, where each of them corresponds to a different genetic group. When animals from different genetic groups mate, their genetic contributions are propagated through the pedigree following the Mendelian rules of inheritance. Offspring in later generations thus inherit different proportions of the genome from the genetic groups. Denote by $q_{ij}$ the expected proportional contribution of base population *j* to the genome of individual *i*. The respective values can be calculated from the pedigree and are typically written into a matrix ${\mathbf {Q}}$ with *n* rows (*n* = number of animals) and *r* columns, such that $q_{ij}$ is the value in the *i*th row and *j*th column (see e.g. Fig. 3 in ref. [17]). Following the notation from Wolak and Reid [17], and denoting by $g_j$ the average genetic effect in group *j*, the basic animal model (1) can be extended to

2 $$\begin{aligned} y_i = \mu + \sum _{j=1}^r q_{ij}g_j + a_i + e_i \ , \end{aligned}$$

where the total additive genetic effect of individual *i* is given as $u_i=\sum _{j=1}^r q_{ij}g_j + a_i$, that is, the weighted sum of genetic group-mean effects, plus the breeding value $a_i$ of the individual that accounts for deviations from the weighted group mean. Note that in model (2) the $q_{ij}$ values play the role of known covariate values (they must be derived from the pedigree before the model is fitted), and the group-specific means $g_j$ are the parameters to be estimated. This model is over-parameterized because for each *i* the contributions from the *r* groups sum up to 1, that is, $\sum _j q_{ij}=1$. Similar to ANOVA models or when categorical variables are included in regression models, the parameters become identifiable when one group is set as reference group (e.g. assuming $g_1=0$), or when additional constraints are added, such as $\sum _j g_j=0$.

Let us illustrate the idea for two genetic groups. When using the convenient constraint $g_1=0$, phenotypes of animals that have ancestors either only from group 1 (i.e. $q_{i1}=1, q_{i2}=0$) or only from group 2 (i.e. $q_{i1}=0, q_{i2}=1$) can be described by the following models

$$\begin{aligned} y_i&= \mu + a_i + e_i&\quad \text {for } i \text { in group 1},\\ y_i&= \mu + g_2 + a_i + e_i&\quad \text {for } i \text { in group 2}. \end{aligned}$$

Thus, members of genetic group 1 have a total additive genetic effect of $u_i = a_i$ and members of genetic group 2 have $u_i = g_2 + a_i$, where $g_2$ estimates the difference between the mean breeding values of the groups. Therefore, the respective $u_i$ values are distributed around an overall mean of 0 and $g_2$, respectively, but with the same $\sigma _A^2$ and relatedness matrix ${\mathbf {A}}$. Note that, while the main benefit of including group-specific means is that bias in the estimates of $\sigma _A^2$ is reduced, the estimated values (e.g. of $g_2$) may sometimes be of interest themselves, as was pointed out previously [17, 22].

### Genetic group models with heterogeneous additive genetic variances

#### Segregation variance for polygenic traits

The key limitation of the genetic group model (2) is that the base populations of all genetic groups are assumed to have the same additive genetic variance, and animal breeders have therefore suggested extensions that allow additive genetic variance to differ among groups, e.g. [19, 24, 33]. However, these methods quickly become computationally demanding because the respective models include terms to account for the segregation variance between any two genetic groups, thus $g(g-1)/2$ segregation variances in the presence of *g* genetic groups. The magnitude of these variances may be considerable in artificial breeding scenarios, for example when crossing genetically differentiated pure-bred lines (see e.g. [35, Table 3]), and if a trait is determined by one or only a few loci. To understand why, let us start by looking at a hypothetical trait that is determined by *m* loci. The segregation variance between two genetic groups (e.g. breeds) can be computed as:

3 $$\begin{aligned} \sigma _S^2 =\frac{1}{2} \sum _{i=1}^m (\alpha _i^c)^2 \end{aligned},$$

[1, equation 9.15], where $\alpha _i^c$ denotes the mean additive genetic difference between the groups due to locus *i*. In an extreme example where only one locus determines a trait value, and where two populations or breeds differ in their mean breeding value by, say, $\alpha ^c=1$, the segregation variance expected in a cross between these breeds is then given as $\sigma _S^2=1/2 \cdot 1 = 0.5$. However, genome-wide association studies (GWAS) suggest that complex (continuous) traits are mostly polygenic, thus the additive genetic component is not determined by a single locus (e.g. [38–41]). In fact, it is a fundamental assumption of quantitative genetics that phenotypic traits are determined by many genes that each contribute a small effect to trait variation, known as the “infinitesimal model” [42, 43]. If, for example, 100 loci each contribute the same proportion of 1/100 to the overall group difference of 1 in the above example, the segregation variance reduces to

$$\begin{aligned} \sigma _S^2 =\frac{1}{2}\cdot 100\cdot (1/100)^2 = 0.005 \ , \end{aligned}$$

which is exactly 1/100 of the segregational variance for a single-locus trait. Thus for any number of loci *m*, the segregation variance is 1 / *m* of what it would be for a single locus, given that each locus contributes the same proportion of the effect. Even when considering that the locus-specific effect sizes are typically heterogeneous, the most influential loci often only explain a small proportion of the phenotypic variance [44–46]. Consequently, the segregation variance $\sigma _S^2$ for complex continuous traits is expected to be small compared to the total phenotypic variance in many study systems [36]. Thus, in the following extension of the animal model, we ignore the segregation variance, and illustrate in our application to the house sparrows that omitting it from the animal model is indeed not critical.

#### Animal model for heterogeneous additive genetic variances

To allow the animal model to account for potentially heterogeneous additive genetic variances of different base populations, we extend model (2) that accounts for group-specific means $g_j$ by splitting the breeding value $a_i$ of each individual *i* into group-specific contributions, similar to [33], such that

4 $$\begin{aligned} y_i&= {} \mu + \sum _{j=1}^r q_{ij}g_j + \sum _{j=1}^r a_{ij} + e_i, \end{aligned}$$

with ${\mathbf {a}}_j^\top = (a_{1j}, \ldots , a_{nj})^\top \sim \mathsf {N}({\mathbf {0}},\sigma _{A_j}^2 {\mathbf {A}}_j)$ for all groups $j=1,\ldots , r$, where $\sigma _{A_j}^2$ is the additive genetic variance in group *j*, and ${\mathbf {A}}_j$ is a group-specific relatedness matrix. We denote $a_{ij}$ as the *partial breeding value*, because it represents the contribution to the breeding value of individual *i* that is inherited from group *j*. Consequently, ${\mathbf {A}}_j$ contains the relatedness at the genes that have come from that group for each pair of individuals. We assume that the contributions $a_{ij}$ of the same individual *i* are independent of each other, because they differ in genetic origin. Importantly, while the proportion of founder genomes $q_{ij}$ are known quantities from the pedigree, the partial breeding values $a_{ij}$ are unknown and must be estimated, which is possible thanks to differences between the ${\mathbf {A}}_j$ matrices. In practice, model (4) may also include fixed effects, like sex or age. We note that the model could easily be extended further to allow for group- or birthplace-specific residual variances, or for group-specific dependencies of the phenotype on fixed effects, namely when the respective effects are expected to differ between environments or genetic groups.

We illustrate the idea of model (4) by again using two genetic groups and assuming $g_1=0$ for identifiability reasons. Animals in genetic group 1 (i.e. $q_{i1}=1$) then have a total additive genetic effect $u_i = a_{i1}$ and animals in genetic group 2 (i.e. $q_{i2}=1$) have $u_i = g_2 + a_{i2}$, whereas the breeding values are distributed as

$$\begin{aligned} (a_{11}, \ldots , a_{n1})^\top&\sim \mathsf {N}({\mathbf {0}},\sigma _{A_1}^2 {\mathbf {A}}_1),\\ (a_{12}, \ldots , a_{n2})^\top&\sim \mathsf {N}({\mathbf {0}},\sigma _{A_2}^2 {\mathbf {A}}_2). \end{aligned}$$

While it is relatively straightforward to formulate such a model, it is less obvious what the group-specific relatedness matrices ${\mathbf {A}}_j$ are. It is, for example, not valid to use ${\mathbf {A}}$ for ${\mathbf {A}}_j$, because the within-group relatedness structure is different from the overall relatedness. In addition, the breeding values are now split into the sum $a_i=\sum _j a_{ij}$, thus $\text {Var}(a_{ij})\le \text {Var}(a_i)$, and $a_{ij}$ equals $a_i$ only if an animal has $q_{ij}=1$ for group *j*. On the other hand, $a_{ij}=0$ if the animal’s genome contains no contribution from the respective group *j* (thus if $q_{ij}=0$). We now turn to the issue of how to obtain the ${\mathbf {A}}_j$ matrices.

#### Group-specific relatedness matrices

*Decomposition of the relatedness matrix* To understand how to specify the group-specific relatedness matrices ${\mathbf {A}}_j$, we will first need to look a little bit deeper into the technical details of how (inverse) relatedness matrices can be efficiently computed. We recall the mathematical approach that Henderson and Quaas proposed to decompose a single population’s relationship matrix ${\mathbf {A}}$ [12, 37], because by understanding the principle, we can derive the group-specific matrices. Henderson and Quaas suggested to decompose ${\mathbf {A}}$ by a *generalized Cholesky decomposition* into

5 $$\begin{aligned} {\mathbf {A}} = {\mathbf {T}} {\mathbf {D}} {\mathbf {T}}', \end{aligned}$$

where ${\mathbf {T}}$ is lower triangular matrix with transposed ${\mathbf {T}}'$, and ${\mathbf {D}}=\text {Diag}(d_{11},\ldots , d_{nn})$ is a diagonal matrix with entries $d_{11}, \ldots , d_{nn}$. A useful property of the decomposition (5) is that the matrices ${\mathbf {T}}$ and ${\mathbf {D}}$ have elegant interpretations: ${\mathbf {T}}$ traces the flow of alleles from one generation to the other, and the diagonal entries of ${\mathbf {D}}$ scale the Mendelian sampling variance [47, p. 27].

**Fig. 1:**
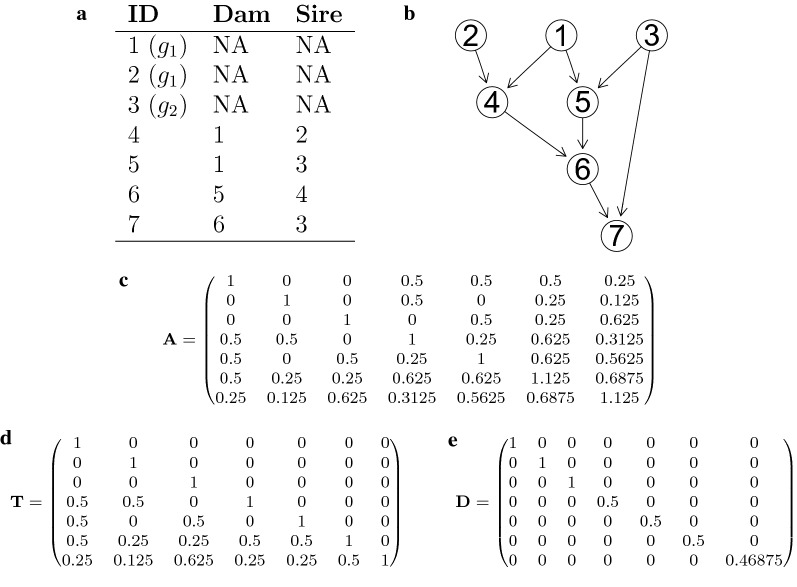
Pedigree example, adapted from Table 2.1 in [47] (**a**). Graphical representation of the pedigree (**b**) with the corresponding relatedness matrix ${\mathbf {A}}$ (**c**). The matrices from the decomposition ${\mathbf {A}}={\mathbf {T}}{\mathbf {D}}{\mathbf {T}}'$ for the genetic groups described in the text are given in (**d**) and (**e**). In the genetic group example, animals 1 and 2 are founders of genetic group 1, and animal 3 of genetic group 2

Let us illustrate these properties with an example that we adapted from Mrode [47, Table 2.1], starting without genetic groups. The pedigree is given in Fig. 1a, with a corresponding graphical representation of parent-offspring relations (Fig. 1b) and matrices ${\mathbf {A}}$, ${\mathbf {T}}$ and ${\mathbf {D}}$ (Fig. 1c–e), where the generalized Cholesky decomposition to obtain ${\mathbf {T}}$ and ${\mathbf {D}}$ was calculated with the function gchol() that is available in the bdsmatrix package in $\mathsf {R}$ [48, 49]. In this example, animals 1, 2 and 3 have phantom parents and are denoted as *founders* of the population. Each off-diagonal entry in ${\mathbf {T}}$ corresponds to the relatedness coefficient (expected relatedness) of individuals with their direct descendants (i.e. children, grandchildren etc.), where columns represent ancestors and rows descendants. For example, individual 1 is the parent of animals 4 and 5, thus the entries (4,1) and (5,1) in the matrix are 0.5. In addition, animal 6 is the offspring of animals 4 and 5, thus the relatedness of 1 with 6 is also 0.5. Finally, the relatedness of 1 and 7 is 0.25. These considerations can be repeated for each column in the matrix, where all diagonal elements are 1 and all elements below the diagonal in the respective column correspond to the *expected* proportion of the genome that is transmitted from the respective ancestor to its direct descendants.

On the other hand, the diagonal entry $d_{ii}$ for animal *i* in ${\mathbf {D}}$ is calculated as

$$\begin{aligned} d_{ii} = \left\{ \begin{array}{@{}l l@{}} 1, &{}\quad \text {if no parent is known},\\ 1- 0.25 - 0.25 (F_{p}), &{}\quad \text {if one parent } p \text { is known},\\ 1 - 0.5 - 0.25 (F_{s} + F_{d}), &{}\quad \text {if both parents } s \text { and } d \text { are known}, \\ \end{array}\right. \end{aligned}$$

where $F_p$, $F_s$ and $F_d$ are the pedigree-based inbreeding coefficients of the known parent(s) [47, p. 28]. We do not index the inbreeding coefficients with the animal identity (*i*) purely for notational simplicity. For later use we note that, in the absence of inbreeding, the diagonal entry is $(1 - 0.5 p_i)$, with $p_i$ corresponding to the proportion of *i*’s parental genome that is known. Possible values are $p_i= 0, 0.5$ or 1 if no, one or two parents are known, respectively. This can be understood as follows: if, for example, one parent of an animal *i* is unknown, its predicted breeding value is 0.5 times the breeding value of the known parent, but the other half of its breeding value is unknown. The deviation from the predicted breeding value that could be obtained if both parents were known is absorbed by the Mendelian sampling deviation. The respective variance thus contains the Mendelian sampling variance *plus* a variance that is due to the unknown parent [50]. The more parents are unknown, the larger is this variance.

*T and D for genetic groups* In the presence of genetic groups, each phantom (i.e. unknown) parent of an observed animal is assigned to one of the groups, and expected proportions of individual’s genomes that originate from the respective genetic groups can be calculated from the pedigree [17, 51]. For simplicity, we again consider the case with two groups, and denote by ${\mathbf {A}}_1$ and ${\mathbf {A}}_2$ the respective relatedness matrices. These can be decomposed in the same way as ${\mathbf {A}}$ into:

6 $$\begin{aligned} {\mathbf {A}}_1&= \mathbf {T_1}\mathbf {D_1} \mathbf {T_1}' \quad \text {and} \end{aligned}$$

7 $$\begin{aligned} {\mathbf {A}}_2&= \mathbf {T_2}\mathbf {D_2} \mathbf {T_2}', \end{aligned}$$

with matrices ${\mathbf {T}}_1$ and ${\mathbf {T}}_2$ describing the transmission of alleles through the generations, and Mendelian sampling variance matrices ${\mathbf {D}}_1$ and ${\mathbf {D}}_2$. The generalization to more than two groups is straightforward.

**Fig. 2:**
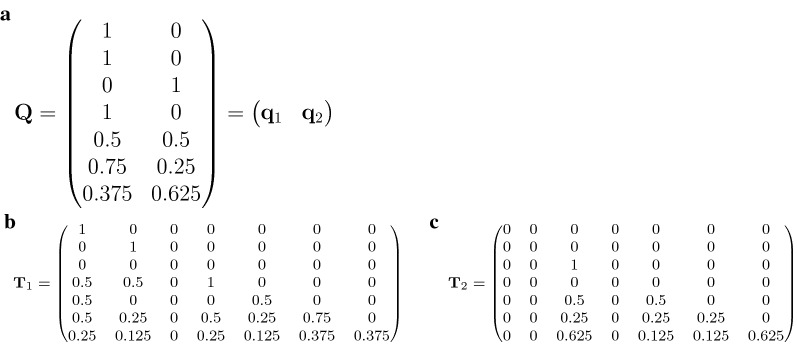
Genetic group matrix ${\mathbf {Q}}$ (**a**), and ${\mathbf {T}}_j$ matrices (**b**, **c**) for the pedigree example. Group-specific proportions of the genome are stored in the ${\mathbf {Q}}$ matrix. The columns of ${\mathbf {Q}}$ can be used to derive the group-wise matrices $\mathbf {T_1}$ and ${\mathbf {T}}_2$ by appropriate multiplication with ${\mathbf {T}}$

Let us assume in the pedigree example of Fig. 1 that the phantom parents of founder animals 1 and 2 belong to genetic group 1, and the phantom parents of animal 3 to genetic group 2. This leads to proportional contributions of each genetic group to the genomes of the descending individuals as given by the matrix ${\mathbf {Q}}$ (Fig. 2a), with column vectors ${\mathbf {q}}_1=(q_{11},q_{21},\ldots ,q_{n1})$ and ${\mathbf {q}}_2 = (q_{12},q_{22},\ldots ,q_{n2})$ that contain the respective proportions of genetic origin from groups 1 and 2 for each individual. The transmission of alleles within each group is represented by the matrices ${\mathbf {T}}_j$ ($j=1,2$). They are designed such that animals with a certain proportion of genetic origin can only pass on the respective fraction of alleles. This means, for example, that an animal *i* with $q_{i1}=0.5$ passes only a proportion of 0.25 (and not 0.5) of alleles to its offspring as part of genetic group 1, while another expected proportion of 0.25 is passed on to its offspring within group 2. The matrices ${\mathbf {T}}_j$ are thus obtained by scaling the respective entries in ${\mathbf {T}}$ by the respective group-proportions. This is achieved by multiplying each row of ${\mathbf {T}}$ by ${\mathbf {q}}_j$ or, equivalently, by

8 $$\begin{aligned} {\mathbf {T}}_j = {\mathbf {T}} \cdot \text {Diag}({\mathbf {q}}_j), \end{aligned}$$

where $\text {Diag}({\mathbf {q}}_j)$ denotes a diagonal matrix with diagonal equal to ${\mathbf {q}}_j$. The matrices ${\mathbf {T}}_1$ and ${\mathbf {T}}_2$ for our example are given in Fig. 2b, c. Note that the diagonal of ${\mathbf {T}}_j$ corresponds to ${\mathbf {q}}_j$, which is the respective expected fraction of the genome that belongs to group *j*, and all entries in the respective column are scaled (i.e. multiplied) with that same value. Animal 5, for example, has $q_{15}=0.5$, thus the fifth column in ${\mathbf {T}}$ is multiplied by 0.5 to obtain the respective column in ${\mathbf {T}}_1$.

Next, we need to find appropriate versions of ${\mathbf {D}}_1$ and ${\mathbf {D}}_2$. We noted in the previous subsection that, in the absence of inbreeding, $d_{ii} = 1 -0.5 p_i$ with $p_i$ representing the proportion of the ancestral genome that is known. To calculate the respective entries $d^{(1)}_{ii}$ and $d^{(2)}_{ii}$ in the group-specific matrices, we have to multiply $p_i$ by the proportions of genetic origin $q_{i1}$ and $q_{i2}$, because the respective product then corresponds to the ancestral proportions that are known *within* the respective group. In the case where only one parent is known, multiplication must be with the genetic proportion of the known parent, denoted here as $q_{ij}^{(p)}$, because only this respective part of the ancestral genome within group *j* is then known. This leads to:

$$\begin{aligned} d^{(j)}_{ii} = \left\{ \begin{array}{@{}l l@{}} 1, &{}\quad \text {if no parent is known}, \\ 1- 0.25 \cdot q_{ij}^{(p)}, &{}\quad \text {if one parent is known},\\ 1 - 0.5 \cdot q_{ij}, &{}\quad \text {if both parents are known}. \\ \end{array}\right. \end{aligned}$$

Let us now also account for inbreeding, which may influence $d^{(j)}_{ii}$ for individuals with at least one parent known. Starting with animals that have both parents known, and rearranging the entries $d_{ii} = 1 - 0.5 - 0.25 (F_s + F_d)$ in the original matrix ${\mathbf {D}}$, leads to

$$\begin{aligned} (F_s + F_d) = 2 - 4 d_{ii} \ . \end{aligned}$$

**Fig. 3:**
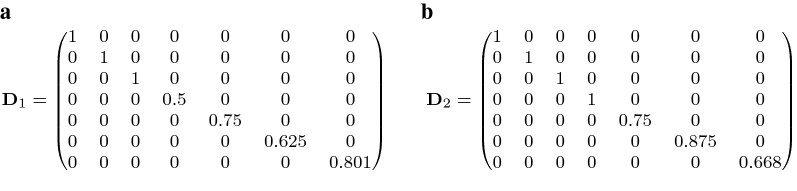
Group-specific matrices ${\mathbf {D}}_1$ and ${\mathbf {D}}_2$ for the example pedigree, derived using the approximation of Eq. (10)

The knowledge of $(F_s + F_d)$ for each animal is useful to derive the entries $d^{(j)}_{ii}$ for group *j* in the presence of inbreeding. By scaling the effect of inbreeding with the group-specific proportions $q_{ij}$ of an animal’s genome, the entries are given as:

9 $$\begin{aligned} d^{(j)}_{ii}&= 1 - 0.5 \cdot q_{ij} - 0.25 \cdot q_{ij} (F_s + F_d) \end{aligned}$$

10 $$\begin{aligned}&= 1 - 0.5 \cdot q_{ij} - 0.25 \cdot q_{ij} (2-4 d_{ii}) \nonumber \\&= 1 - q_{ij} (1 - d_{ii}), \end{aligned}$$

where the third line is an algebraic simplification of the second line. The same calculation for animals with only one parent known, using $d_{ii}=1-0.25-0.25(F_p)$ and solving for $F_p$, leads to the same formula with $q_{ij}$ replaced by $q_{ij}^{(p)}$ from the known parent. Finally, if both parents are unknown (i.e. for $d_{ii}=1$), the formula also leads to the correct value of $d^{(j)}_{ii}=1$. Applying formula (10) to the above pedigree example yields ${\mathbf {D}}_1$ and ${\mathbf {D}}_2$ (Fig. 3a, b).

Formula (10) is simple and convenient. However, it provides only an approximation of the correct matrix entries, because in (9) we assumed that parental inbreeding can simply be scaled by the genetic group proportions $q_{ij}$ (for two known parents) or $q_{ij}^{(p)}$ (for one known parent). Instead, the theoretically correct way to deal with parental inbreeding coefficients to derive $d_{ii}^{(j)}$ would be to use the actual *partial* (i.e. group-specific) parental inbreeding coefficients, denoted e.g. as $F_s^{(j)}$ or $F_d^{(j)}$ for parents *s* and *d*. These group-specific inbreeding coefficients contain only the inbreeding that emerges due to inbreeding *within genetic group* *j*, that is, they measure the probability that an individual is identical by descent for an allele that descended from founders within group *j* [52]. The correct way to calculate $d_{ii}^{(j)}$ is thus given by:

11 $$\begin{aligned} d^{(j)}_{ii} = \left\{ \begin{array}{@{}l l@{}} 1, &{}\quad \text {if no parent is known}, \\ 1- 0.25 \cdot q_{ij}^{(p)} - 0.25 (F_p^{(j)}), &{}\quad \text {if one parent } p {\text{ i }s known},\\ 1 - 0.5 \cdot q_{ij} - 0.25 (F_s^{(j)} + F_d^{(j)}), &{}\quad \text {if both parents } s\hbox { and } d \hbox { are known}. \\ \end{array}\right. \end{aligned}$$

Obviously, this formula requires the calculation of group-specific inbreeding coefficients, which is computationally cumbersome. One way to obtain these coefficients is by first calculating *founder*-specific inbreeding coefficients that partition the total inbreeding coefficient $F_i$ into the additive components $F_{ik}$ from each founder animal *k*, as proposed by Lacy et al. [52]. Because partial contributions for all founders sum up to $F_i$ (e.g. [52, 53]), we can sum only over founders from genetic group *j* to obtain group-specific inbreeding coefficients $F_i^{(j)} = \sum _{k \in \text {group }j} F_{ik}$.

Let us illustrate the difference between the approximate method suggested in Eq. (10) and the correct formula for $d^{(j)}_{ii}$ given in Eq. (11) for our example from Fig. 1. Animal 6 is the only parent in the pedigree with a non-zero inbreeding coefficient, which is $F_6=0.125$. However, because animals 1 and 2 are founders of group 1 and animal 3 is a founder of group 2, the pedigree reveals that inbreeding originates only from matings within group 1. Therefore, $F_6$ is split into group-specific inbreeding coefficients as $F_6^{(1)}=0.125$ and $F_6^{(2)}=0$. By plugging these values into Eq. (11) to estimate the respective values for animal 7 (which is the only animal that is affected by this change), we obtain $d_{7,7}^{(1)}=0.812$ and $d_{7,7}^{(2)}=0.656$, which are quite close to the approximate values $d_{7,7}^{(1)}=0.801$ and $d_{7,7}^{(2)}=0.668$ from Fig. 3a, b. Note that, in this paper, we will continue to use the convenient and computationally efficient approximation of Eq. (10) to scale the entries in ${\mathbf {D}}_j$, but we will illustrate the consequences of this approximation in Additional file 1.

*Properties of group-specific relatedness matrices* Once the components ${\mathbf {T}}_j$ and ${\mathbf {D}}_j$ for each group *j* are known, a simple matrix multiplication yields the group-specific relatedness matrices ${\mathbf {A}}_j = {\mathbf {T}}_j {\mathbf {D}}_j {\mathbf {T}}_j'$. Remembering that ${\mathbf {T}}_j = {\mathbf {T}} \cdot \text {Diag}(q_{1j},\ldots ,q_{nj})$, ${\mathbf {D}}_j = \text {Diag}(d^{(j)}_{11},\ldots , d^{(j)}_{nn})$ with entries derived from Eqs. (10) or (11), and that the product of diagonal matrices is obtained by component-wise multiplication of the diagonal entries, this expression can be simplified to

12 $$\begin{aligned} {\mathbf {A}}_j = {\mathbf {T}} {\tilde{\mathbf {D}}}_j {\mathbf {T}}', \end{aligned}$$

with ${\tilde{\mathbf {D}}}_j$ representing the diagonal matrix with entries $q_{ij}^2 d_{ii}^{(j)}$ for $i=1,\ldots ,n$. Consequently, the population-specific ${\mathbf {T}}$ can be used for each group, and we only need to calculate the group-specific diagonal elements of ${\tilde{\mathbf {D}}}_j$ to derive ${\mathbf {A}}_j$ for each group *j*.

**Fig. 4:**
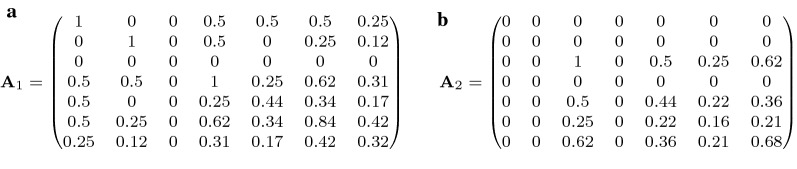
Group-specific relatedness matrices for the example pedigree with entries rounded to two digits

The ${\mathbf {A}}_j$ matrices for the two groups considered in our example are given in Fig. 4. An important aspect is that both ${\mathbf {A}}_1$ and ${\mathbf {A}}_2$ contain columns and rows with all variances and covariances equal to zero, namely for animals *i* without a contribution from group *j* ($q_{ij}=0$). While this is theoretically correct, because the respective breeding value is then $a_{ij}=0$, the resulting matrices are singular. When it comes to implementation, the problem can be solved by replacing zeros on the diagonal by very small values, for example $10^{-6}$ or even $10^{-12}$. The choice is not critical in our experience, but for deeper pedigrees we recommend smaller values, because the relatedness of the most distant relatives in the pedigree may then also become very small. In any case, it would be prudent to check the robustness of the results to different values.

*Scaling the inverse relatedness matrix* In practice, it is usually the inverse relatedness matrix, not ${\mathbf {A}}$, that is required to fit the models, thus ${\mathbf {A}}^{-1}$ is typically calculated and stored directly from the pedigree. Consequently, we also need to derive the inverse group-specific matrices ${\mathbf {A}}_j^{-1}$. From Eq. (5) it follows that the inverse of ${\mathbf {A}}$ can be decomposed into ${\mathbf {A}}^{-1} = ({\mathbf {T}}^{-1})' {\mathbf {D}}^{-1} {\mathbf {T}}^{-1}$, where ${\mathbf {D}}^{-1} = \text {Diag}(1/d_{11},\ldots , 1/d_{nn})$. Using Eq. (12), we see that

13 $$\begin{aligned} {\mathbf {A}}^{-1}_j = ({\mathbf {T}}^{-1})' {\tilde{\mathbf {D}}}_j^{-1} {\mathbf {T}}^{-1}, \end{aligned}$$

thus it is sufficient to derive ${\mathbf {T}}^{-1}$ and ${\mathbf {D}}^{-1}$ directly from the pedigree, and to calculate ${\tilde{\mathbf {D}}}_j^{-1}$ with diagonal entries $1/(d_{ii}^{(j)} q_{ij}^2)$ for each group (see Additional file 2 for a coded $\mathsf {R}$ example). Alternatively, if ${\mathbf {A}}^{-1}$ is already known, ${\mathbf {T}}^{-1}$ and ${\mathbf {D}}^{-1}$ can be obtained from a generalized Cholesky decomposition, although this might be computationally less convenient. Again, entries with $q_{ij}=0$ are replaced by very small values, e.g. $10^{-12}$, to avoid singularities.

## Simulation

### Generating data

To illustrate the performance of genetic group models with group-specific additive genetic variances, we simulated data using the simGG() function from the $\mathsf {R}$ package nadiv [54]. The function allows the generation of pedigrees and phenotypes for a focal population (group 1) that receives a specified number of immigrants from another population in each generation (group 2). Group-specific mean breeding values and additive genetic variances can be set by the user, and breeding values for the founder animals of both genetic groups are sampled from the respective distributions. Offspring breeding values are calculated from the parental mean, plus a Mendelian sampling deviation that depends on the additive genetic variance of the resident population, but there is no additional term that induces a segregation variance (for more details see [54]). The simulation assumes random mating among individuals that currently live in the same population, thus offspring may inherit genetic components from both genetic groups due to immigration. The contributions $q_{ij}$ from group *j* for animal *i* were calculated with the ggcontrib() function from the nadiv package. For data generated with group-specific mean breeding values and additive genetic variances, the appropriate underlying model for the analysis is:

14 $$\begin{aligned}&y_i = \mu + q_{i2}g_2 + a_{i1} + a_{i2} + e_i, \nonumber \\&{\mathbf {a}}_1^\top \sim \mathsf {N}({\mathbf {0}},\sigma _{A_1}^2 \mathbf {A_1}), \quad {\mathbf {a}}_2^\top \sim \mathsf {N}({\mathbf {0}},\sigma _{A_2}^2 \mathbf {A_2}), \nonumber \\&e_i \sim \mathsf {N}(0,\sigma _E^2), \end{aligned}$$

where we used the same notation as in Eq. (4) and fixed the focal group mean $g_1=0$ for identifiability reasons [thus the term $q_{i1}g_1$ is omitted from Eq. (14)]. We simulated data according to three different scenarios, but always setting the population mean $\mu =10$, group means $g_1=0$, $g_2=2$, group-specific additive genetic variances $\sigma _{A_1}^2=2$ and $\sigma _{A_2}^2=3$ and residual variance $\sigma _E^2=1$. Each scenario encompassed 10 non-overlapping generations.

**Scenario 1** The carrying capacity of the population was set to 300 individuals. In each generation, 100 mating pairs were created by random sampling with replacement from the adults, and each pair contributed 4 offspring to the next generation. In addition, 30 immigrants were added to the focal population in each (except the first) generation. Finally, a subset of the offspring was randomly selected such that the population size always corresponded exactly to its carrying capacity.

**Scenario 2** This scenario was the same as scenario 1, except that only 5 (instead of 30) immigrants were allowed in each of the non-overlapping generations, so that animals of the immigrant group were rare.

**Scenario 3** In this scenario we used the same carrying capacity as above, 20 immigrants per generation, but we only allowed for 5 breeding pairs per generation that produced 60 offspring each. While this scenario has an immigration rate that lies between scenarios 1 and 2, the small number of breeding pairs induces higher inbreeding levels. This scenario is therefore suitable to illustrate the consequence of using approximation (10) to scale the group-specific Mendelian sampling variance matrices ${\mathbf {D}}_j$, because the approximation affects the scaling of parental inbreeding coefficients and is thus most relevant in the presence of inbreeding.

For each scenario, we generated 100 datasets, and each of them was analyzed with the genetic group animal model that accounted for heterogeneous additive genetic variances $\sigma _{A_1}^2$ and $\sigma _{A_2}^2$, as given in Eq. (14). In addition, we compared the results to the outcome of the standard genetic groups model that allowed for different mean breeding values, but only a single (homogeneous) variance in both groups, given as:

15 $$\begin{aligned}&y_i = \mu + q_{i2}g_2 + a_i + e_i, \nonumber \\&{\mathbf {a}}^\top \sim \mathsf {N}({\mathbf {0}},\sigma _A^2 {\mathbf {A}}), \quad e_i \sim \mathsf {N}(0,\sigma _E^2)\ . \end{aligned}$$

For scenario 3 we also investigated how close the group-specific Mendelian sampling variance approximations for $d_{ii}^{(j)}$ from Eq. (10) are in comparison to the correct version given in Eq. (11). The group-specific inbreeding coefficients present in the correct formula were calculated with the software GRain [55] (details are given in Additional file 1). Correlation coefficients $\rho$ between the (correct) $d_{ii}^{(j)}$ values from Eq. (11) and the approximated values from formula (10) were calculated, and all simulations were analyzed with both versions for comparison.

Following the recommendation by He and Hodges [56], we stored posterior modes (and not posterior means) of the variance components in each iteration. All models were fitted with integrated nested Laplace approximations (INLA, version from June 20, 2017) using the $\mathsf {R}$ interface R-INLA, which provides a fast and accurate alternative to MCMC [57], although it has so far only rarely been used for animal models (but see e.g. [58, 59]). All variance components were given penalized complexity (PC) priors [60], which were suggested as robust alternatives to the popular but criticized gamma priors [61]. The $\text {PC}(u,\alpha )$ prior has an intuitive parameterization: The prior probability for the standard deviation $\sigma$ is given as $\Pr (\sigma >u)=\alpha$ (with $0< \alpha <1$). More information and plots that compare various gamma to PC priors can be found in Additional file 1. Here we used $\text {PC}(1,0.05)$ priors for all variances (thus $\Pr (\sigma >1)=0.05$ *a priori*), but results were insensitive to this choice. All fixed effect parameters were assigned independent $\mathsf {N}(0,10^4)$ priors. A short tutorial including $\mathsf {R}$ code to generate and analyze data using integrated nested Laplace approximations (INLA) and MCMCglmm [62] for the models used here can be found in Additional file 2.

### Simulation results

**Fig. 5:**
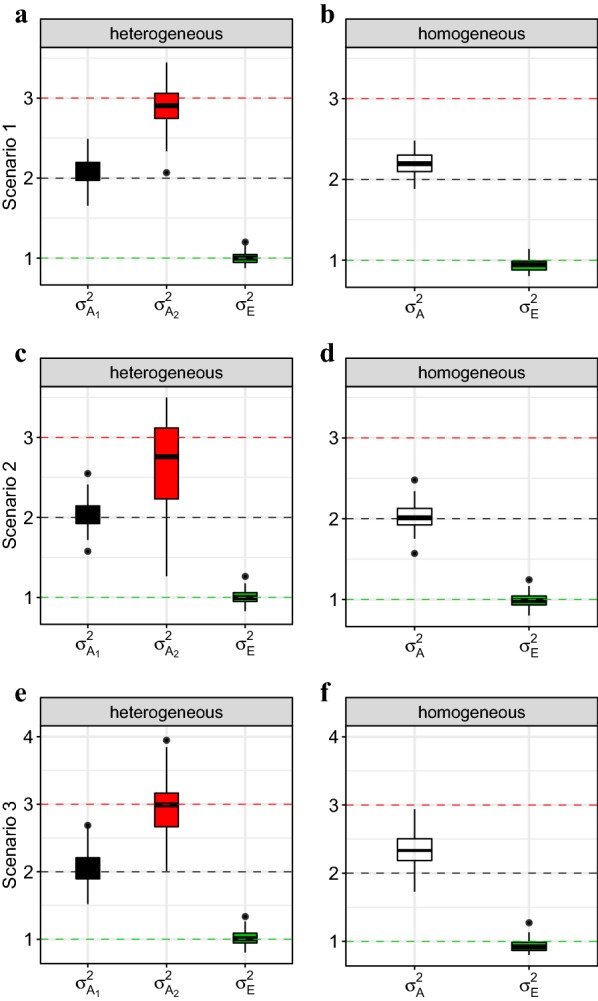
Results from 100 iterations for simulation scenarios 1–3. The boxplots represent the distributions of estimated variances (posterior modes) from a model with genetic groups and heterogeneous additive genetic variances $\sigma _{A_1}^2$ and $\sigma _{A_2}^2$ (left panel), compared to the results from a model that only allowed for a single homogeneous variance $\sigma _{A}^2$ (right panel). Dashed lines indicate the reference values that were used to generate the data (black: $\sigma _{A_1}^2$, red: $\sigma _{A_2}^2$, green: $\sigma _{E}^2$)

Estimates from model (14) with correctly specified heterogeneous group variances were close to the variances used to generate the data in scenarios 1 and 3, while the model estimates in scenario 2 suffer from large uncertainty (Fig. 5, left). In particular the variance $\sigma _{A_2}^2$ of the underrepresented immigrant population in scenario 2 was difficult to identify and biased towards $\sigma _{A_1}^2$ (Fig. 5c). Thus, the results indicate that the genetic group model (14) is able to isolate approximately correct group-specific additive genetic variances, but that some caution is required if representatives of a genetic group are rare in the dataset. When the data were analyzed with the genetic group model (15) that included a single variance $\sigma _A^2$, the variance estimate usually laid between the two simulated group-specific variances (Fig. 5, right). In the presence of only few immigrants, the estimate tended to be close to $\sigma _{A_1}^2$ (scenario 2), while more immigration (scenarios 1 and 3) caused the estimate of $\sigma _A^2$ to be intermediate between $\sigma _{A_1}^2$ and $\sigma _{A_2}^2$, although still closer to $\sigma _{A_1}^2$. This is as expected, and illustrates that genetic group models with a single, homogeneous variance will estimate a value in between the true group-specific additive genetic variances, with a tendency towards the variance of the more numerous group. These patterns were qualitatively similar when the additive genetic variances of the immigrant and resident population were switched, such that residents had larger additive genetic variance than immigrants (results not shown).

Simulation scenario 3 led to datasets with a mean inbreeding coefficient of $F=0.10$. Interestingly, the comparison between the approximate versus the correct values in the ${\mathbf {D}}_1$ and ${\mathbf {D}}_2$ matrices shows that the approximation suggested in Eq. (10) leads to $d_{ii}^{(j)}$ values that are highly correlated with the correct values from Eq. (11). As an example, we found correlation coefficients $\rho \ge 0.988$ in three randomly selected simulation runs. In addition, using the correct ${\mathbf {D}}_j$ matrices led to distributions of estimated variances that are indistinguishable from the results when the approximations were used (see Additional file 1: Figures S1 and S2 ).

## Application: house sparrow data

### Study population

As a proof of concept, we applied our method to empirical data from a long-term study of an insular house sparrow meta-population off the Helgeland coast in northern Norway. The study has been running continuously since 1993, and is used as a model system to examine ecological and evolutionary processes in fragmented vertebrate populations, e.g. [63–65]. The islands in the meta-population differ in characteristics related to environmental conditions, habitat type and population size, with considerably larger and more stable populations on the five islands that are located closer to the mainland (denoted as *inner* islands) compared to the three islands located further away (denoted as *outer* islands). The ten remaining islands are summarized as *other* islands (see in Additional file 1: Figure S4 for an overview of the island system).

Small blood samples were collected from all captured birds on the eight inner and outer islands to provide DNA for single nucleotide polymorphism (SNP) genotyping on a 200K SNP array, see [41]. Only successfully genotyped birds that were also measured for body mass and/or wing length were included in our animal model analyses. For inner and other islands, the dataset included phenotypic measurements taken during the breeding seasons since 1993 and 1995, respectively. Due to strong population bottlenecks on the outer islands in 2000 [65], only measurements taken since 2002 were used for the populations in the outer group. Details on how morphological measurements of wing length and body mass were obtained on adult birds are given by e.g. [63, 66].

Parentage analyses for the eight island populations in this study were carried out with the $\mathsf {R}$ package SEQUOIA [67]. Briefly, SNP genotype data of all adults recorded as present on any of the eight inner or outer islands during the years 1998-2013 (the inner group) or 2003-2013 (the outer group) were used in the parentage analyses. This resulted in a “meta-population pedigree” ($N = 3116$) spanning up to 14 generations (Niskanen et al., personal communication), where both parents were known for 52.7%, one parent was known for 25.0%, and no parent was known for 22.3% of the individuals. Since SEQUOIA introduces *dummy parents* to preserve known relationships, e.g. sibling relationships, even when parents are not genotyped, a higher percentage of individuals had “known” parentages (81.0%, 5.5% and 13.5% with two, one or no parents known, respectively).

The genetic group analysis that we carry out here requires that each unknown (i.e. phantom) parent must be assigned to one of the base populations, that is, we must attribute phantom parents to the *inner*, *outer* or *other* island group to determine their genetic origin. This was done here by first identifying the natal (hatch) island of all individuals, either from ecological data or, if unavailable, by using genetic assignment procedures based on the SNP genotype data (Saatoglu et al., personal communication). Of all individuals in our dataset, 2012, 640, and 93 individuals were assigned to a natal island in the inner, outer and other group, respectively. This information was then used to assign phantom parents to the genetic group to which the hatch island of the respective individual belonged. As an example, if an individual with phantom parents is known to be born on one of the inner islands, the respective phantom parents were assumed to belong to the base population of the inner island group, and so on. The relatively high connectivity of the local pedigrees on the islands, with a total of 22.5% migrants between islands, and 8.9% migrants between the island groups (i.e. between inner, outer and other islands, Saatoglu et al., personal communication), helps minimizing bias due to common environment effects in our analyses. Finally, because inbreeding depression is known to occur in our study system [68], we accounted for any inbreeding effects on body mass and wing length by including each individual’s genomic inbreeding coefficient $F_{\text {GRM}}$ [8, 69] as a covariate in all models fitted here.

### Analysis of wing length and body mass

With all phantom parents assigned to one of the genetic groups (*inner*, *outer*, *other*), each individual *i* obtained expected proportions of genetic origin $q_{ij}$ from the three groups *j* by propagating the founder individual’s genome through the pedigree using the ggcontrib() function. Note that the different groups are very unequal in sample size, which can be seen from the summation of genetic proportions over all animals within a group *j*, that is, $n_j = \sum _i q_{ij}$, which corresponds to equivalents of full animal genomes (Table 1). This is not surprising given the smaller population sizes [65], lower recapture rates [70], and shorter time-series for populations in the outer island group, and considering that there were no systematic genotyping efforts on the other islands. Thus, we only see genomes from the other islands if they were introduced via immigration events to one of the inner or outer islands.

For the two traits investigated here, mass (in g) and wing length (in mm), we fitted separate models that accounted for sex (0 = males, 1 = females), inbreeding ($F_{\text {GRM}}$), month of measurement (numeric with values 5, 6, 7, and 8) and age (in years) as fixed effects that were stored in matrix ${\mathbf {X}}$. Furthermore, current island of residence where the measurement was taken (*island*), hatch year (*year*), animal (*id*) and an independent residual term (*e*) were included as random factors. An individual *i* was included in the model if it had at least one recorded observation *k* of the respective trait. The model with group-specific mean and variances of the breeding values is thus given as:

16 $$\begin{aligned} y_{ik} = \mu + {\mathbf {X}}_i {\varvec{\upbeta }} + \underbrace{\sum _{j=2}^3 q_{ij} g_{j} + \sum _{j=1}^3 a_{ij}}_{u_i} + island_{ik} + year_{i} + id_i + e_{ik}, \end{aligned}$$

where the total genetic contribution $u_i$ is the sum of the weighted means $g_j$ and the variability from the different genetic groups, as introduced in Eq. (4), and ${\varvec{\upbeta }}$ is the vector of fixed effects. The three genetic groups *inner*, *outer* and *other* are encoded as groups 1, 2 and 3, respectively, where the mean of the inner group was set to $g_1=0$ for identifiability reasons. Thus, the estimates $g_2$ and $g_3$ reflect differences in group means with respect to the inner populations. The components $a_{i1}$, $a_{i2}$ and $a_{i3}$ are distributed with mean zero and heterogeneous $\sigma _{A_1}^2$, $\sigma _{A_2}^2$ and $\sigma _{A_3}^2$, with dependency structures given by the group-specific relatedness matrices ${\mathbf {A}}_1$, ${\mathbf {A}}_2$ and ${\mathbf {A}}_3$ that were calculated as explained in the Methods section.

The results from fitting model (16) were compared to the standard genetic group model that only accounts for differences in group means, but with homogeneous $\sigma _A^2$ and dependency structure defined through the relatedness matrix ${\mathbf {A}}$. Both models were fitted to the data using INLA. All variances were given $\text {PC}(1,0.05)$ priors, and fixed effects parameters were assigned independent $\mathsf {N}(0,10^4)$ priors. Interestingly, the results indicate that the outer group has a somewhat larger estimated additive genetic variance than the inner group for both traits (Table 1), although the respective 95% credible intervals (CI) are large and overlap. We therefore also calculated the posterior distribution of the differences between these variances by relying on an R-INLA specific function inla.hyperpar.sample() that allows to efficiently draw a large number of samples from the joint posterior distribution of a model. For body mass, the difference $\sigma _{A_1}^2 - \sigma _{A_2}^2$ between inner and outer additive genetic variances obtained from 100, 000 samples had a mode of $- 0.67$, with a 95% CI ranging from $- 1.81$ to 0.22, whereas for wing length the mode was $- 0.31$, with a 95% CI ranging from $- 1.50$ to 0.27 (the full distributions of the differences are given in Additional file 1: Figure S5 ). Thus, there is some weak indication that the additive genetic variances for wing length and body mass might actually differ between the two island groups.

While it is not possible to draw strong conclusions about group-specific differences in the additive genetic variances from the data at hand, the results could still indicate that animals living on distinct island groups differ in the level of variation in the genes that affect some phenotypic traits, e.g. due to different allele frequencies. Although additional shared environmental effects might also be confounded with estimates of group-specific additive genetic variances [7], dispersal among local sparrow populations helps minimizing (yet cannot fully eliminate) such problems. In both cases, the additive genetic variance estimates from the homogeneous model are rather close to the estimates for the inner base population, which is expected because the latter is by far the largest genetic group. For comparison, we also calculated the observed phenotypic variance ${\hat{\sigma }}_P^2$ of the two traits, where the respective group-specific phenotypic variances were only calculated using the pure-bred animals (i.e. those with $q_{ij}=1$) in each group (Table 1). In addition, the estimates for $g_2$ indicate that animals on the outer islands have, on average, a lower additive genetic value, although the evidence is weak in the case of wing length (Table 2). The direction of these effects is in agreement with earlier findings regarding the phenotype of the birds, which indicate that individuals on the outer islands are lighter and have shorter wings than individuals on the inner islands [71, 72]. The residual variance estimates are not of primary interest, and they are all very similar between the models with heterogeneous and homogeneous additive genetic variances. The respective results are therefore given in Additional file 1: Table S3 .

All results presented here involved the approximate approach from formula (10) to scale the group-specific ${\mathbf {D}}_j$ matrices. To illustrate that this approximation is unproblematic, we repeated all calculations with the correct versions as given in Eq. (11), which again involved the gene dropping method provided by the GRain program. Details are given in Additional file 1, and all results remain essentially unchanged (see in Additional file 1: Figure S3 and Tables S1 and S2). Finally, it is worth reiterating that model (16) does not account for the three segregation variances that would occur between any pair of groups, because these are expected to be negligibly small, but also because estimating these three additional variances would impose unrealistic requirements on these data. To illustrate that ignoring segregation variances is not critical, we also fitted a model with a segregation term for the sparrow example with only two genetic groups (inner and outer). Details on how to estimate segregation variances are given in Additional file 1. Importantly, Table S4 confirms that the segregation term between these two groups is indeed very small (with posterior modes $<0.001$ for both traits), and that its inclusion only leads to irrelevant changes in the results.

**Table 1:** Estimates (posterior modes; posterior means) and 95% CI (defined as the 2.5 to 97.5% quantile intervals, given in parentheses) of the three group-specific additive genetic variances (${\hat{\sigma }}_A^2$) for inner, outer and other genetic groups, as well as for a single homogeneous variance across groups (total) for body mass and wing length

	Body mass			Wing length		
	${\hat{\sigma }}_{A}^2$	$n_j$ or *n*	${\hat{\sigma }}_{P}^2$	${\hat{\sigma }}_{A}^2$	$n_j$ or *n*	${\hat{\sigma }}_{P}^2$
Inner	1.41; 1.47 (1.07, 1.98)	1490.4	5.77	1.76; 1.83 (1.53, 2.23)	1487.3	5.30
Outer	2.10; 2.21 (1.32, 3.32)	352.6	7.92	1.96; 2.20 (1.56, 3.24)	349.1	6.85
Other	0.34; 0.76 (0.12, 2.17)	128.0	3.98	1.28; 1.54 (0.79, 2.80)	126.6	5.69
Total	1.56; 1.59 (1.21, 2.05)	1971	5.53	1.79; 1.86 (1.59, 2.21)	1963	5.41

The sample sizes denote the equivalent of full animal genomes that are present in the three genetic groups ($n_j$, for the model with heterogeneous variances) or in the total dataset for the respective trait (*n*, for the homogeneous model). For comparison, the phenotypic variances (${\hat{\sigma }}_P^2$) of the total population and the three groups are given, where the group-specific versions were calculated only from the 992, 144 and 50 pure-bred animals in the inner, outer and other groups

**Table 2:** Posterior means and 95% CI of the fixed effects for the animal models used for body mass and wing length

	Body mass		Wing length	
	Heterogeneous	Homogeneous	Heterogeneous	Homogeneous
Sex (females)	0.47	0.48	$- 2.76$	$- 2.77$
	(0.29, 0.64)	(0.30, 0.65)	($- 2.89$, $- 2.63$)	($- 2.90$, $- 2.63$)
$F_{GRM}$	$-\, 1.13$	$-1.27$	$- 1.35$	$- 1.38$
	($- 3.00$, 0.73)	($- 3.12$, 0.58)	($- 2.73$, 0.02)	($- 2.75$, $- 0.01$)
Month	$- 0.30$	$- 0.30$	$- 0.19$	$- 0.19$
	(− 0.36, − 0.24)	($-0.36$, $- 0.24$)	($- 0.22$, $- 0.15$)	($- 0.22$, $- 0.15$)
Age	0.08	0.08	0.47	0.47
	(0.02, 0.14)	(0.02, 0.14)	(0.43, 0.50)	(0.43, 0.50)
$g_2$ (outer)	$- 0.45$	$- 0.43$	$- 0.15$	$- 0.26$
	($- 0.84$, $- 0.06$)	($- 0.87$, 0.02)	($- 0.48$, 0.18)	($- 0.65$, 0.14)
$g_3$ (other)	$- 0.36$	$- 0.32$	$- 0.18$	$- 0.22$
	($- 0.83$, 0.10)	($- 0.89$, 0.26)	($- 0.55$, 0.18)	($- 0.70$, 0.27)

The estimates were extracted from models with either group-specific (heterogeneous) or a single (homogeneous) additive genetic variance

## Discussion

We have introduced an extension of the animal model that allows for unequal additive genetic variances in the presence of multiple interbreeding genetic groups. Our method decomposes the breeding value $a_i$ of each individual *i* into contributions $a_{ij}$ from each group *j*. These so-called partial breeding values are assumed to covary according to group-specific relatedness matrices ${\mathbf {A}}_j$. To understand how the respective matrices are constructed, we rely on the Cholesky decomposition of the full relatedness matrix ${\mathbf {A}}$ into components ${\mathbf {T}}$ and ${\mathbf {D}}$ [12], and show how simple algebraic scaling operations can be used to derive the group-specific versions ${\mathbf {T}}_j$ and ${\mathbf {D}}_j$, which are then again multiplied to obtain ${\mathbf {A}}_j$. We also discuss how, in practice, the inverses ${\mathbf {A}}_j^{-1}$ can be derived using the same theoretical framework. The method is computationally efficient, in particular when an (accurate) approximation for the group-specific Mendelian sampling variance matrices ${\mathbf {D}}_j$ is used, and because we omit any segregation variance terms from the models. Although genetic group animal models have been used before, in particular in animal and plant breeding setups, modeling heterogeneous additive genetic variances has so far been considered unfeasible for natural study systems [17]. Therefore, while natural populations have previously (although still rarely) been analyzed with genetic group models that account for mean differences in additive genetic effects [21, 22], to the best of our knowledge such populations have never been analyzed with animal models that account for heterogeneous variances. In principle, the variance estimates could also be used to assess group-specific versions of heritability [3] or evolvability [73], where $\sigma _A^2$ is replaced by the group-specific version $\sigma _{A_j}^2$.

We have assumed that segregation variances are often negligible for polygenic traits. How often will this assumption hold? The segregation variance from formula (3) approximates zero whenever the *infinitesimal model*, which underlies the animal model and which posits that traits are determined by a large number of genes with small effects, holds approximately. This is likely the case for many polygenic, complex traits [74]. Our estimates of the segregation variance in the empirical house sparrow dataset support the view that segregation variance may often be negligible. This result is mirrored in GWAS of the genetic architectures of body mass, wing length and other morphological traits in house sparrows and other passerines, which revealed a polygenic basis, where any significant genomic region explains only a very small proportion of the phenotypic variance [40, 41]. Taken together, these results suggest that segregation variances can often be neglected in genetic group models, provided the traits of interest are truly very polygenic. When focal traits have a genetic architecture with only few causal genes with a large effect, omitting the segregational variance may however introduce a non-negligible bias in the estimated additive genetic variances. In such a case, it is still possible to formulate a model that accounts for segregation variances, as explained in Additional file 1, although such models may quickly impose unrealistic demands on the data.

Estimating and disentangling individual variance components is generally known to be difficult, and it is particularly challenging for genetic group models with group-specific additive genetic variances. The problem is that the group-specific covariance matrices ${\mathbf {A}}_j$ are the sole sources of information for the discrimination of the variance components, yet these matrices may be similar in the presence of many animals with mixed group ancestry. The results from the house sparrow example in Table 1 illustrate that group-specific variance estimates suffer from larger uncertainty than a single homogeneous variance, especially when group sizes are relatively small, as is the case for the *outer* and *other* groups. On the other hand, computation remains feasible thanks to the small size of the groups and the total size of the pedigree. We thus face an intrinsic trade-off of many estimation procedures, where more data are needed to fit more complex models reliably, but the latter may in turn become computationally prohibitive.

An obvious “objective” way to decide whether extending the animal model to heterogeneous additive genetic variances is needed might be via the use of information criteria, such as the deviance information criterion for Bayesian models [75]. However, we do not recommend to rely on the DIC (for some DIC criticism see e.g. [76]), nor on any other “automatic” way to do model selection. A particular difficulty with model selection in our context is that the heterogeneous and homogeneous models are *linearly equivalent* [77], that is, both models lead to the same marginal model and therefore to the same log-likelihood. Consequently, it is not possible to distinguish between model fits in a likelihood framework (e.g. using an AIC criterion), and differences in DIC may simply stem from the estimation of the effective number of parameters, which depends on the priors. Therefore, the decision to model heterogeneous additive genetic variances should be based on biological knowledge, and in particular when interest explicitly centers around group-specific variances. Posteriors of the differences among additive genetic variances, which we have calculated for the sparrow application, may inform about the biological relevance of these differences.

The reason why additive genetic variances of the sparrows’ body mass and wing length tend to be larger for the outer group compared to those for the inner group is not directly obvious, but assuming the pattern is real, one can speculate whether some kind of interaction between genetic drift and dispersal is involved. Low effective population size ($N_e$) is expected to increase the rate of genetic drift and reduce $\sigma _A^2$ [78]. Because populations in the outer group have lower $N_e$ than populations in the inner group [65], the outer populations could be expected to have a smaller additive genetic variance than the inner populations. However, two processes may have counteracted this loss of additive genetic variance due to genetic drift. First, the population bottlenecks and subsequent inbreeding that occurred on the outer islands around 2000 may actually have increased additive genetic variance [79] in the outer compared to the inner populations. And second, because the outer populations experience higher dispersal rates than the inner populations, outer populations consist of a larger proportion of immigrants from the inner populations than vice versa ([64] and Saatoglu et al., personal communication), and dispersal among populations may increase additive genetic variance in the direction of divergence [80], this could have resulted in a tendency for larger additive genetic variance in the outer group compared to the inner group. However, further studies are needed to tease apart these alternative explanations.

A general limitation of genetic group models is that parent-offspring relations are needed to propagate genetic contributions from founder individuals through the pedigree to determine ${\mathbf {Q}}$. Despite the optimism of letting genomic relatedness matrices take over for pedigrees [14], these alone are unfortunately not sufficient to fit genetic group models. A combination of genomic and pedigree information, the latter possibly inferred from genetic data [67, 81], may however provide a powerful basis to overcome this limitation. In addition, genomic data can provide valuable information about the ancestry of founder individuals, and enable (proportional) genetic assignment of these individuals to different genetic groups, e.g. [82, 83].

## Conclusions

The proposed extension of genetic group models will be useful for any study population that is structured into subpopulations, given that sufficient information on dispersal or crossbreeding events is available. In particular, the fact that group-specific additive genetic variances can be estimated for subpopulations that are not completely isolated might also be useful when interest centers around the dependency of additive genetic variance on the effective population size, a relation that is of pivotal interest in evolutionary and conservation biology. Finally, the method may provide a starting point to assess temporal or spatial variation of additive genetic variance, for example by defining populations at certain time points as base populations.

## Additional files

**Additional file 1.** Additional theory and supplementary figures. Document including additional plots and derivations.

**Additional file 2.** Coded example. Short tutorial including R code and explanations on how to simulate data with genetic groups, and how to fit a genetic group model with heterogeneous variances using INLA and MCMCglmm.
