# Supplementary material for: Animal models with group-specific additive genetic variances: extending genetic group models
Source: Genet Sel Evol. 2019 Feb 28;51:7. doi: 10.1186/s12711-019-0449-7 (PMC6394059; doi:10.1186/s12711-019-0449-7)
Supplement: Supplementary file 1 — Additional file 1. Additional theory and supplementary figures. Document including additional plots and derivations. [file 12711_2019_449_MOESM1_ESM.pdf]

Additional file 1 for

Animal models with group-specific additive genetic variances: extending genetic group models

Stefanie Muff[1,2*], Alina K. Niskanen[3,4], Dilan Saatoglu[3], Lukas F. Keller[1,5], Henrik Jensen[3]

February 1, 2019

[1] Institute of Evolutionary Biology and Environmental Studies, University of Zurich, Winterthurerstrasse 190, Zurich, Switzerland

[2] Epidemiology, Biostatistics and Prevention Institute, Department of Biostatistics, University of Zurich, Hirschengraben 84, Zurich, Switzerland

[3] Centre for Biodiversity Dynamics, Department of Biology, Norwegian University of Science and Technology, Høgskoleringen 5, Trondheim, Norway

[4] Department of Ecology and Genetics, University of Oulu, Oulu, Finland

[5] Zoological Museum, University of Zurich, Karl-Schmid-Strasse 4, Zurich, Switzerland

1 Partial inbreeding coefficients and correct scaling of the D matrix

1.1 Calculation of partial inbreeding coefficients

As briefly described in the main text, pedigree-based inbreeding coefficients F_i can be decomposed into partial inbreeding coefficients F_{ik}, which measure the probability that an individual is homozygous (identical by descent, IBD) for an allele that descended from founder k (Lacy *et al.*, 1996; Gulisija *et al.*, 2006). Thanks to the fact that the total inbreeding coefficient is the sum of all partial inbreeding coefficients, $F_i = \sum_k F_{ik}$, summation over founder individuals from a (genetic) group j allows to derive group-specific inbreeding coefficients, that is

$$F_i^{(j)} = \sum_{k \in \text{group } j} F_{ik} \ .$$

To obtain the founder-specific inbreeding coefficients we used the software `GRain` (Baumung *et al.*, 2015). In brief, the procedure uses a stochastic gene dropping approach that assigns two unique alleles to each founder and generates the genotypes of all descendants along the actual pedigree following Mendelian segregation rules. This gene dropping process is repeated a large number of times, and the inbreeding coefficient F_i is estimated as the proportion of IBD loci out of all loci in the genome of individual i. `GRain` stands for "Genetic Rain", and it is able to calculate several versions of ancestral inbreeding coefficients, such as those one originally proposed by Ballou (1997). Here, we used `GRain` only to derive the founder-specific partial inbreeding coefficients F_{ik} that sum up to the total F_i for each individual, as proposed by Lacy *et al.* (1996). The program is written in Fortran90, but we used the R interface (`GRain4R`) that is provided by the authors.

As described in the main text, the correct way to calculate the entries in the group-specific Mendelian sampling variance matrices $\mathbf{D}_j$ is given by

$$d_{ii}^{(j)} = \begin{cases} 1 \ , & \text{if no parent is known,} \\ 1 - 0.25 \cdot q_{ij}^{(p)} - 0.25(F_p^{(j)}) \ , & \text{if one parent } p \text{ is known,} \\ 1 - 0.5 \cdot q_{ij} - 0.25(F_s^{(j)} + F_d^{(j)}) \ , & \text{if both parents } s \text{ and } d \text{ are known ,} \end{cases} \quad (1)$$

where $q_{ij}^{(p)}$ is the genetic group proportion of animal i's parent p in group j (if only one parent is known), q_{ij} is the respective value for individual i, and $F_p^{(j)}$, $F_s^{(j)}$ and $F_d^{(j)}$ are the group-specific parental inbreeding coefficients. We refer to the matrices $\mathbf{D}_j$ calculated by definition (1) as the *correct* version, in contrast to the *approximate* version derived from formula (10) in the main text.

1.2 Using correct $\mathbf{D}$ matrices in the simulation study (scenario 3)

For each of the 100 iterations of scenario 3 in the simulation study, we calculated the group-specific inbreeding coefficients with `GRain`, where we used 10 000 stochastic gene dropping iterations in each case. For computational efficiency, we calculated group-specific inbreeding coefficients only for the resident group 1 (*i. e.* $F_i^{(1)}$ for all animals i) and then derived the respective values for the immigrants by $F_i^{(2)} = F_i - F_i^{(1)}$. This is possible because the group-specific inbreeding coefficients for each animal i must sum up to total inbreeding coefficient F_i (Lacy *et al.*, 1996; Baumung *et al.*, 2015). Correct group-specific $\mathbf{D}$ matrices were then derived by using these group-specific inbreeding coefficients of the respective parents, as derived in equation (1) above (which corresponds to equation (11) in the main text). To give an impression of how good the approximate $\mathbf{D}_j$ are in comparison to the correct version, Figure S1 shows the approximate against the correct entries d_{ii}^j for groups 1 and 2 for three randomly selected simulation runs.

In each of the 100 simulation iterations we then also fitted the animal model with heterogeneous variances using the correct $\mathbf{D}_j$ matrices. The distribution of the estimated variances is given in Figure S2, and the results are almost identical to those from the approximate version given in Figure 5 of the main text. The large correlations indicated in Figure S1 and the good agreement between variances estimated with the correct and approximate methods illustrate that the approximation to the $\mathbf{D}_j$ matrices by formula (10) in the main text are reliable.

1.3 Using correct $\mathbf{D}$ matrices in the house sparrow example

We also ran `GRain` for 100 000 gene dropping iterations to obtain founder-specific inbreeding coefficients for the house sparrow example. Again, because computations become inefficient for large sets of founder individuals, we carried out these iterations only for the two smaller groups of *outer* and *other* founders, derived group-specific inbreeding coefficients by summing over all founders within these groups, and then obtained the respective values for the inner group by $F_i^{(inner)} = F_i - F_i^{(outer)} - F_i^{(other)}$.

The group-specific inbreeding coefficients were then used to obtain correct versions of $\mathbf{D}_1$, $\mathbf{D}_2$ and $\mathbf{D}_3$ for the inner, outer and other group, respectively. For all three matrices, we plotted the approximate against the correct versions of $d_{ii}^{(j)}$, see Figure S3. All correlations were at least 0.998, indicating that the computationally more convenient approximation of the $\mathbf{D}_j$ matrices is not critical. This is also confirmed by the results from the animal models: Additive genetic variances for the correct model are given in Table S1, and the estimates are almost identical to those from the approximate approach (Table 2 in the main text).

	Body mass	Wing length
inner ($\hat{\sigma}^2_{A_1}$)	1.38 (1.06, 1.98)	1.76 (1.53, 2.24)
outer ($\hat{\sigma}^2_{A_2}$)	2.10 (1.34, 3.34)	1.96 (1.55, 3.23)
other ($\hat{\sigma}^2_{A_3}$)	0.43 (0.16, 2.52)	1.27 (0.80, 2.85)

Table S1: Estimates (posterior modes) and 95% CIs (in parentheses) of the three group-specific additive genetic variances ($\hat{\sigma}^2_A$) for inner, outer and other genetic groups for the house sparrows example when the "correct" scaling of the $\mathbf{D}_j$ matrices according to equation (11) of the main text is used. The results show only minor differences with respect to those given in Table 2 of the main text, where the approximate scaling of equation (10) was used.

	Body mass	Wing length
sex (females)	0.47	−2.76
	(0.29, 0.64)	(−2.89, −2.63)
F_{GRM}	−1.13	−1.36
	(−3.00, 0.74)	(−2.74, 0.03)
month	−0.30	−0.19
	(−0.36, −0.24)	(−0.22, −0.15)
age	0.08	0.47
	(0.02, 0.14)	(0.43, 0.50)
g_2 (outer)	−0.45	−0.15
	(−0.84, −0.06)	(−0.47, 0.19)
g_3 (other)	−0.36	−0.18
	(−0.83, 0.10)	(−0.55, 0.18)

Table S2: Posterior means and 95% CIs of the fixed effects for the animal models of the house sparrows when correct $\mathbf{D}_j$ matrices were used.

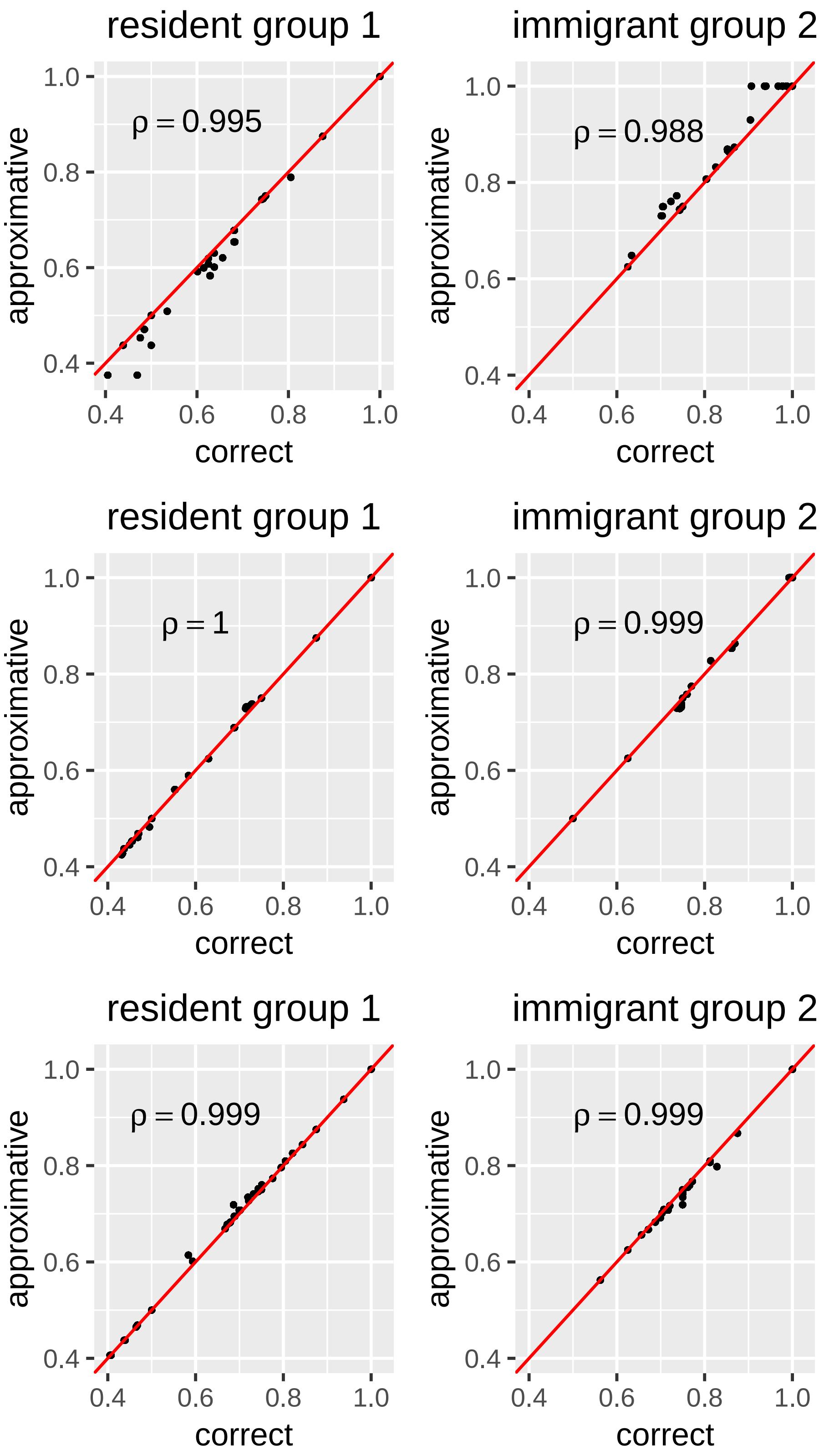

Figure S1: Comparison of approximate against correct scaling of group-specific $d_{ii}^{(j)}$ entries of the $\mathbf{D}_1$ and $\mathbf{D}_2$ matrices for three randomly selected runs of simulation scenario 3. The Pearson correlation ρ between the respective d_{ii} values of the two approaches is given, and the red line is the $y = x$ reference.

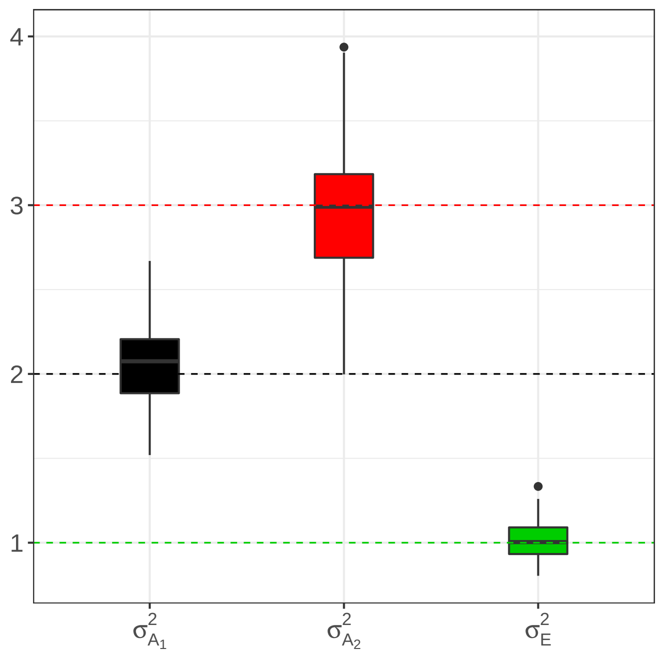

Figure S2: Results from 100 iterations for simulation scenarios 3 when the correct versions of $\mathbf{D}_1$ and $\mathbf{D}_2$ were used. The boxplots represent the distributions of estimated variances (posterior modes) from a model with genetic groups and heteroscedastic additive genetic variances $\sigma^2_{A_1}$ and $\sigma^2_{A_2}$. Dashed lines indicate the reference values that were used to generate the data (black: $\sigma^2_{A_1}$, red: $\sigma^2_{A_2}$, green: σ^2_E).

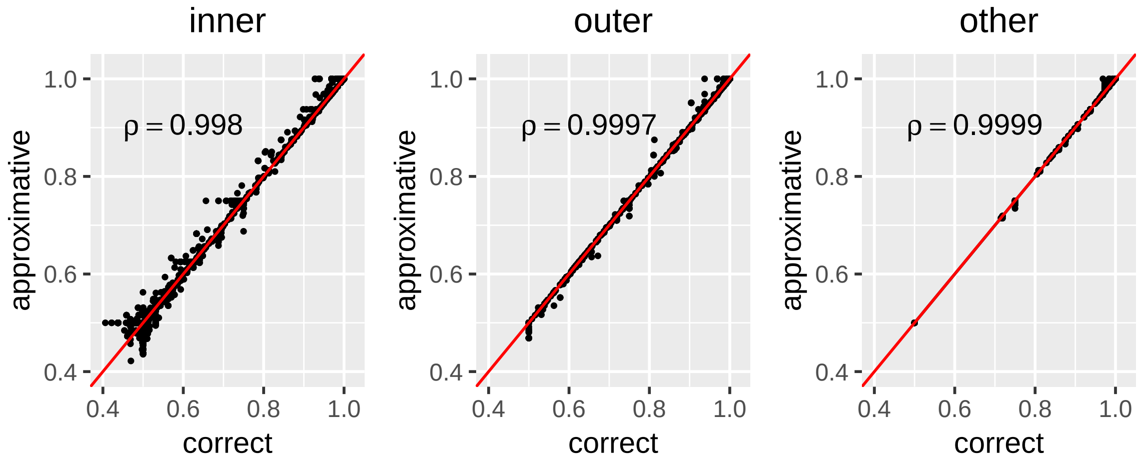

Figure S3: Comparison of approximate against correct scaling of group-specific $d_{ii}^{(j)}$ entries of the $\mathbf{D}_j$ matrices for the house sparrow example. The correlation coefficient ρ is given for the three groups (inner, outer, other), and the red lines denote the $y = x$ reference.

2 Supplementary information about the house sparrows application

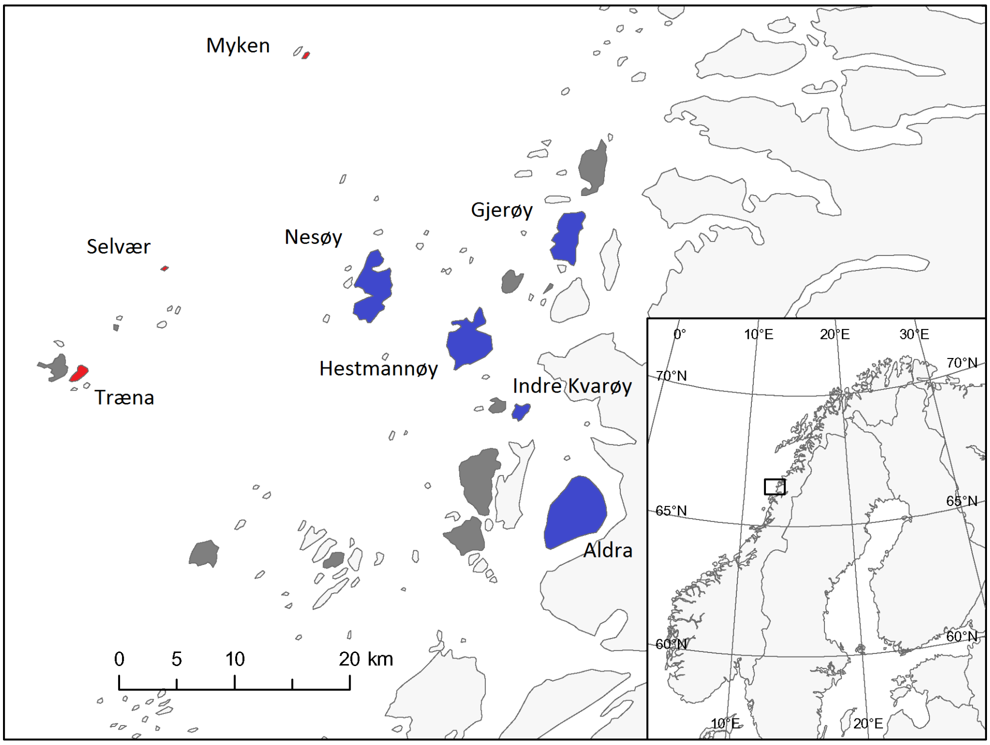

Figure S4: Map showing the insular house sparrow metapopulation off the Helgeland coast in northern Norway. The eight island populations used in the current study are named. Myken, Selvær, and Træna belong to the *outer* group (shown in red), whereas Gjerøy, Nesøy, Hestmannøy, Indre Kvarøy and Aldra belong to the *inner* group (shown in blue). The remaining 10 islands which had a house sparrow population for at least one year during the long-term study (starting in 1993) belong to the *other* group and are shown in dark grey. Further islands in the system (without sparrows) and the mainland (with a few, small house sparrow populations) are shown in light grey.

	Body mass		Wing length	
	Heterogeneous	Homogeneous	Heterogeneous	Homogeneous
σ^2_{island}	0.06 (0.02, 0.43)	0.07 (0.03, 0.54)	0.07 (0.03, 0.40)	0.08 (0.04, 0.41)
σ^2_{year}	0.05 (0.01, 0.15)	0.05 (0.009, 0.13)	0.04 (0.009, 0.11)	0.04 (0.01, 0.12)
σ^2_{id}	1.02 (0.77, 1.42)	0.90 (0.66, 1.32)	0.34 (0.20, 0.52)	0.27 (0.14, 0.45)
σ^2_{e}	2.87 (2.71, 3.03)	2.86 (2.72, 3.04)	1.00 (0.92, 1.04)	1.00 (0.91, 1.04)

Table S3: Estimates (posterior modes) and 95% CIs (in parentheses) for the random effects variances for current island, hatch year, animal id and residual variance, both for models with heterogeneous or homogeneous additive genetic variances.

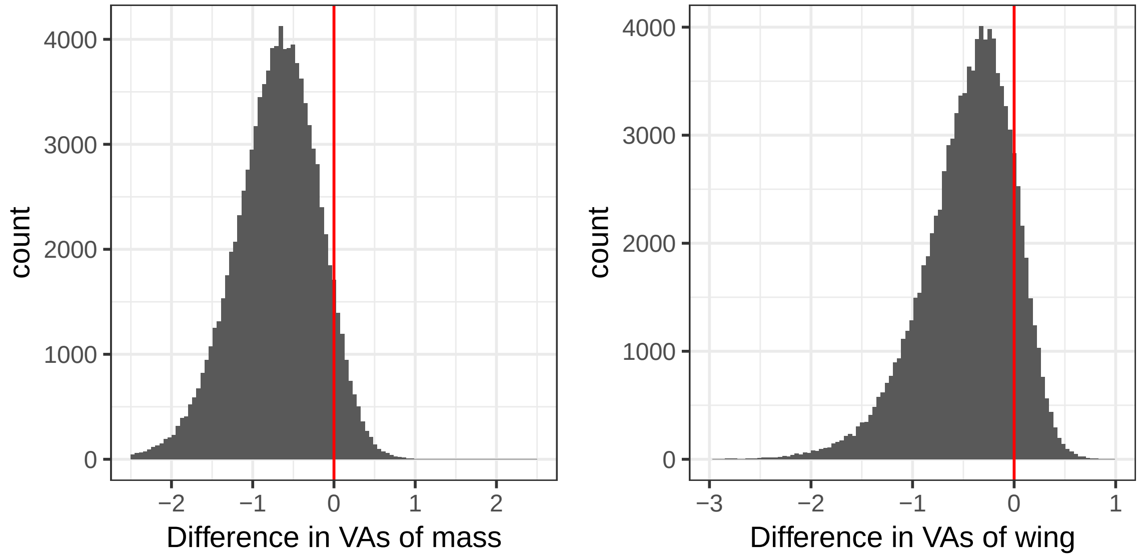

Figure S5: Distribution of 100 000 samples from the posterior distribution of the differences $\sigma^2_{A_1} - \sigma^2_{A_2}$ for body mass (left) and wing length (right) in the house sparrow application. The dashed red line indicates a difference of zero.

3 Accounting for segregation variance in the house sparrow example

In all genetic group animal models considered so far we assumed that segregation variance terms are "small" and can thus be neglected. Nevertheless, for illustration we describe here how to fit a model that accounts for segregation variance, and confirm that the segregation variance is very small in the house sparrow example. Segregation terms occur between any *pairs* of genetic groups (*e. g.* Lo *et al.*, 1993; García-Cortés and Toro, 2006), thus in the presence of g genetic groups, a total number of $g\cdot(g-1)/2$ segregation variances needs to be estimated. The three genetic groups in the house sparrow data would thus require that three segregation variances are estimated, on top of the three additive genetic variances. This however imposes unrealistic requirements on these data. In order to make the estimation of variances feasible, we therefore use a model with only two genetic groups (inner and outer), which requires only the inclusion of one segregation variance (between the inner and outer groups), on top of the two group-specific additive genetic variances. Animals from the *other* group were thereby included in the inner group, because other and inner groups are relatively similar with respect to their mean breeding values for both traits (see Table 3 of the main text).

Using model (4) from the main text for two groups requires that we add an additional random term for the segregation variance. For animal i this term is denoted as $s_i^{(12)}$, thus

$$y_i \;=\; \mu + q_{i2}g_2 + a_{i1} + a_{i2} + s_i^{(12)} + e_i \ , \tag{2}$$

with $\boldsymbol{s}^{(12)} \sim \mathsf{N}(\mathbf{0}, \sigma^2_{s_{12}}\mathbf{A}_{12})$ and covariance matrix $\mathbf{A}_{12}$. The remaining components of the model are the same as before, that is $\boldsymbol{a}_1^\top \sim \mathsf{N}(\mathbf{0}, \sigma^2_{A_1}\mathbf{A}_1)$ and $\boldsymbol{a}_2^\top \sim \mathsf{N}(\mathbf{0}, \sigma^2_{A_2}\mathbf{A}_2)$, and g_2 is the difference in the mean breeding values between the two groups.

What remains is to derive the covariance matrix $\mathbf{A}_{12}$ for the segregation variance between groups 1 and 2. To this end, we again need to appropriately scale the Cholesky factors $\mathbf{T}$ and $\mathbf{D}$. Using equation (26) from Lo *et al.* (1993), the scaling constants are now given as $c_i^{(12)} = 2\cdot(q_{i1}^S q_{i2}^S + q_{i1}^D q_{i2}^D)$, where *e. g.* q_{i1}^S and q_{i1}^D are the genetic group proportions of the individual i's sire (S) and dam (D) for group 1, respectively. Using the same rationale as for the group-specific matrices, and using vector notation $\boldsymbol{c}^{(12)} = (c_1^{(12)}, \ldots, c_n^{(12)})$ we obtain $\mathbf{T}_{12}$ by scaling

$$\mathbf{T}_{12} = \mathbf{T}\cdot \mathrm{Diag}(\boldsymbol{c}^{(12)}) \ . \tag{3}$$

To obtain the diagonal entries in $\mathbf{D}_{12}$, we use formula (10) of the main text, but replace q_{ij} by $c_i^{(12)}$

$$d_{ii}^{(12)} = 1 - c_i^{(12)}(1 - d_{ii}) \ .$$

These matrix components are then multiplied to obtain the covariance matrix $\mathbf{A}_{12} = \mathbf{T}_{12}\mathbf{D}_{12}\mathbf{T}_{12}'$. The same considerations as in the section *Scaling the inverse relatedness matrix* in the main text illustrate that the inverse can be derived as

$$\mathbf{A}_{12}^{-1} = (\mathbf{T}^{-1})'\tilde{\mathbf{D}}_{12}^{-1}\mathbf{T}^{-1} \ , \tag{4}$$

with diagonal matrix $\tilde{\mathbf{D}}_{12}^{-1}$ with diagonal entries $1/\left(d_{ii}^{(12)}\left[c_i^{(12)}\right]^2\right)$. Again, entries with $c_i^{(12)} = 0$ may occur for individuals with $q_{i1} = 0$ or 1, thus $c_i^{(12)} = 0$ are replaced by very small values, *e. g.* 10^{-12}, to avoid singularities.

Table S4 contains the results for models without and models with the segregation term for mass and wing length. Clearly, the segregation variances (posterior modes) are extremely small, where the relatively large right ends of the 95% CI are due to boundary effects and illustrate the difficulty to reasonably estimate the segregation variance. Importantly, the posterior distributions of the VAs are almost unaffected by the inclusion of the segregation term, confirming that the segregation variance can be safely ignored in the system studied here.

	Body mass		Wing length	
	Without σ_s^2	With σ_s^2	Without σ_s^2	With σ_s^2
$\hat{\sigma}_{A_1}^2$	1.38 (1.06, 1.97)	1.42 (1.08, 1.95)	1.78 (1.55, 2.20)	1.78 (1.55, 2.20)
$\hat{\sigma}_{A_2}^2$	2.10 (1.37, 3.39)	2.04 (1.33, 3.36)	2.35 (1.65, 3.09)	2.35 (1.63, 3.08)
$\hat{\sigma}_{s_{12}}^2$		$5e-05$ ($2e-04$, 0.54)		$2e-04$ ($3e-04$, 0.66)

Table S4: Estimates (posterior modes) and 95% CIs (in parentheses) for the additive genetic variances without and with the inclusion of the segregation variance term.

Will the small segregation variances induce a sampling covariance among the VAs, if they are omitted from the model? If the infinitesimal model does approximately hold, and segregation terms are very small, we do not expect this to be a relevant problem. In fact, the respective correlations and covariances derived from the sparrow models with two groups indicated no increase in sampling correlations when the segregation term were omitted, as illustrated in the results given in Table S5. To obtain these results, we derived 100 000 samples from the joint posteriors of the fitted models, using the `inla.hyperpar.sample()` function from the `R-INLA` package, both for the cases with and without segregation variance. Interestingly, the correlations and covariances among the group-specific VAs were small for all models. In the case of body mass, the model with the segregation variance even had an almost 50% larger

correlation, which is contrary to our expectations. The reasons for this can be manyfold, for example because some of the founder individuals were assigned to a wrong natal island, or because the *other* group was merged with the *inner*, whereas some of the other islands could be more similar to the outer group due to their geographic proximity (see also Figure S4). In any case, the example indicates that increased correlations between VAs when segregation terms are omitted are probably not practically relevant.

	Body mass		Wing length	
	Without σ_s^2	With σ_s^2	Without σ_s^2	With σ_s^2
$\text{Cor}(\sigma^2_{A_1}, \sigma^2_{A_2})$	0.16	0.18	0.07	0.08
$\text{Cov}(\sigma^2_{A_1}, \sigma^2_{A_2})$	0.02	0.02	0.005	0.005

Table S5: Sampling correlations (Cor) and covariances (Cov) between the VAs of inner and outer populations, estimated from 100 000 posterior samples.

4 Penalized complexity priors

For all analyses carried out here we use penalized complexity (PC) priors on the precisions $\tau = 1/\sigma^2$ (Simpson *et al.*, 2017). Note that in Bayesian statistics, we typically work with precisions instead of variances, and we adhere to this convention, especially because INLA also deals with precisions. PC priors were suggested as robust alternatives to the gamma priors, whereas the latter have been critiziced in the past (see *e.g.* Lambert *et al.*, 2005). One problem with gamma priors is that distributions like $\text{Gamma}(\text{shape} = \varepsilon, \text{rate} = \varepsilon)$, with $\varepsilon = 0.01$ or 0.001, are often used in the absence of prior knowledge, because these are assumed to be uninformative. However, this is not the case. As an example, Hodges (2014, p.33) points out that the 95% percentile of the $\text{Gamma}(0.001, 0.001)$ distribution is $3 \cdot 10^{-20}$, thus 95% of the prior weight lies on values between 0 and $3 \cdot 10^{-20}$. Obviously, this is anything but uninformative. We can easily check this using R:

```
qgamma(0.95,shape=0.001,rate=0.001)

## [1] 2.974e-20
```

The situation is a bit better with $\text{Gamma}(0.01, 0.01)$:

```
qgamma(0.95,shape=0.01,rate=0.01)

## [1] 0.3363
```

But even then only a very small fraction of the prior weight on the variances, which then follow an inverse gamma distribution with the same parameters, lies in the range of realistic values. This can be seen by calculating the prior probability that is given to variances below a certain value of the variance, for example 10:

```
library(actuar)
pinvgamma(10,shape=0.01,scale=0.01,lower.tail=TRUE)

## [1] 0.06143
```

The calculation shows that very little prior weight is assigned to variances below 10. This is not surprising, given that the respective gamma distribution for the precision τ puts a lot of prior weight on small precisions, which correspond to large variances. On the other extreme, the `R-INLA` default prior on precisions corresponds to $\text{InvGamma}(1, 0.00001)$ priors on the variances, and these assign almost all the weight to very small variances. We can, for example, verify that more than 99.9% of the prior weight is assigned to $\sigma^2 < 0.01$:

```
pinvgamma(0.01,shape=1,scale=0.00001,lower.tail=TRUE)

## [1] 0.999
```

On the other hand, with PC(u, α) priors the analyst can steer how much prior weight is assigned to certain standard deviations (σ) – not variances – below a certain threshold. The prior probability for the standard deviation σ is given as $\mathsf{Pr}(\sigma > u) = \alpha$ (with $0 < \alpha < 1$). As an example, the PC$(1, 0.1)$ distribution assigns exactly 10% of the weight to $\sigma > 1$, that is, $\Pr(\sigma > 1) = 0.1$ and $\Pr(\sigma \leq 1) = 0.9$, thus the prior probability of σ values below 1 is 0.9. The philosophy behind these priors is related to the Occam's razor principle, which says that models should not be made more complex than necessary, thus the largest portion of prior weight should be assigned to simpler "reference" or "zero" models. Two PC distributions are graphically compared to two common gamma distributions in Figure S6, both transformed to the scale of the variances for better comparison (thus the respective inverse gamma distributions are shown). Although it is difficult to see, we know (from above) that InvGamma$(1, 0.00001)$ assigns almost all the weight to very tiny variances, whereas InvGamma$(0.01, 0.01)$ assigns most weight to large variances. On the other hand, the two PC priors assign a predefined proportion of the prior weight to variances below a certain limit, and successively penalize larger values. By changing the u and α values, the user can easily and intuitively control the distribution.

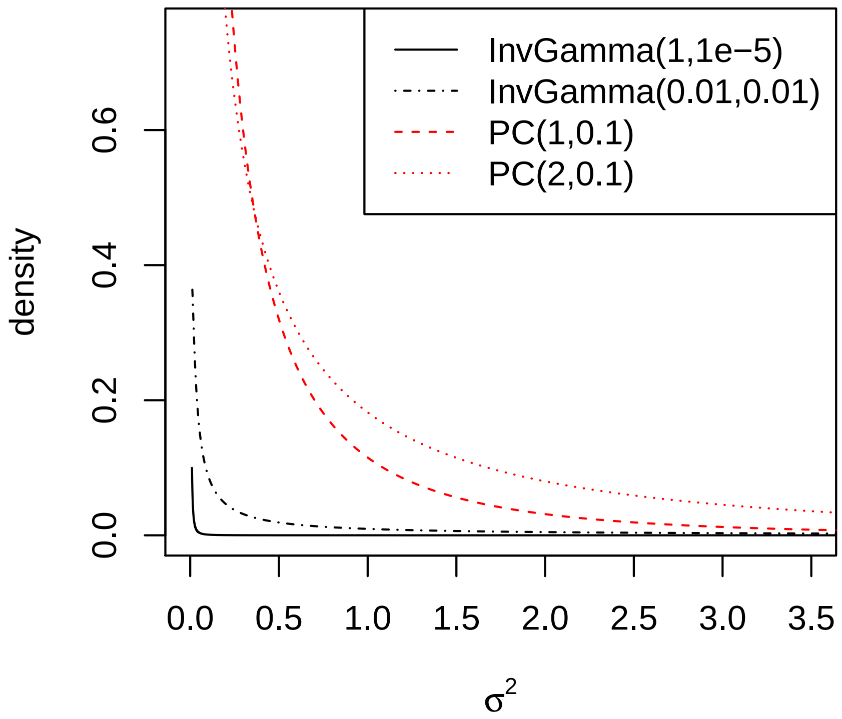

Figure S6: Comparison of the inverse gamma priors and the novel PC priors. Both distributions are shown for the variances, thus inverse gamma (instead of gamma) distributions are used.

References

Ballou, J. D. (1997). Ancestral inbreeding only minimally affects inbreeding depression in mammalian populations. *Journal of Heredity*, **88**, 169–178.

Baumung, R., Farkas, J., Boichard, D., Mészáros, G., Sölkner, J., and Curik, I. (2015). GRAIN: a computer program to calculate ancestral and partial inbreeding coefficients using a gene dropping approach. *Journal of Animal Breeding and Genetics*, **132**, 100–108.

García-Cortés, L. A. and Toro, M. Á. (2006). Multibreed analysis by splitting the breeding values. *Genetics Selection Evolution*, **38**, 601.

Gulisija, D., Gianola, D., Weigel, K., and Toro, M. (2006). Between-founder heterogeneity in inbreeding depression for production in jersey cows. *Livestock Science*, **104**, 244 – 253.

Hodges, J. S. (2014). *Richly Parameterized Linear Models: Additive, Time Series, and Spatial Models Using Random Effects*. Chapman & Hall/CRC, Boca Raton.

Lacy, R. C., Alaks, G., and Walsh, A. (1996). Hierarchical analysis of inbreeding depression in peromyscus polionotus. *Evolution*, **50**, 2187–2200.

Lambert, P., Sutton, A., Burton, P., Abrams, K., and Jones, D. (2005). How vague is vague? a simulation study of the impact of the use of vague prior distributions in MCMC using WinBUGS. *Statistics in Medicine*, **24**, 2401–2428.

Lo, L. L., Fernando, R. L., and Grossman, M. (1993). Covariance between relatives in multibreed populations: additive model. *Theoretical and Applied Genetics*, **87**, 423–430.

Simpson, D., Rue, H., Riebler, A., Martins, T. G., and Sørbye, S. H. (2017). Penalising model component complexity: A principled, practical approach to constructing priors. *Statistical Science*, **32**, 1–28.
