# Supplementary material for: Animal models with group-specific additive genetic variances: extending genetic group models
Source: Genet Sel Evol. 2019 Feb 28;51:7. doi: 10.1186/s12711-019-0449-7 (PMC6394059; doi:10.1186/s12711-019-0449-7)
Supplement: Supplementary file 2 — Additional file 2. Coded example. Short tutorial including R code and explanations on how to simulate data with genetic groups, and how to fit a genetic group model with heterogeneous variances using INLA and MCMCglmm. [file 12711_2019_449_MOESM2_ESM.pdf]

Additional file 2 to "Animal models with group-specific additive genetic variances: extending genetic group models"

By S. Muff, A.K. Niskanen, D. Saatoglu, L.F. Keller, H.Jensen (2018)

R version 3.4.4 (2018-03-15)

Author: Stefanie Muff

Description

We provide code to derive group-specific relatedness matrices following the theory from the main text, and analyze data with INLA.

We will generate data with genetic group effects following the description in Wolak and Reid (2017), Appendix 6. The `simGG()` function from the nadiv R package allows to generate phenotypic and pedigree data for a resident and an immigrant population. In each generation, a specified number of immigrants is added to a focal population. The function allows to set group-specific additive genetic variances. Please consult `?simGG()` for more explanation.

Generating data

Start by loading some packages

```
library(INLA)
library(Matrix)
library(MCMCglmm)
library(nadiv)
library(pedigreemm)
library(bdsmatrix)

set.seed(197863)
```

Set additive genetic variance of resident and immigrant populations

```
varf <- 2
vari <- 3
```

Set the environmental variance

```
vare <- 1
```

Generate g=10 generations with 300 animals per generations, 100 pairs and 4 offspring per pair, and 30 immigants per generation (except in the first generation):

```
ggHetVar <- simGG(K = 300, pairs = 100, noff = 4, g = 10,
                  nimm = 30,
                  muf = 0, # mean breeding value of residents
                  mui = 2, # mean breeding value of immigrants
                  mup = 10, # mean of population
                  VAf = varf, VAi = vari,
                  VRf = vare, VRi = vare
    )
```

Check the data structure and rename a bit

```
head(ggHetVar)
```

```
##   id dam sire parAvgU mendel           u           r         p      pred.u
## 1  1  NA   NA      NA     NA  2.34890397  1.36207078 13.710975  2.48833175
## 2  2  NA   NA      NA     NA  0.70595843  1.19940286 11.905361  1.28458945
## 3  3  NA   NA      NA     NA -2.10756996  0.69372635  8.586156 -0.92821383
## 4  4  NA   NA      NA     NA  2.46450334  1.33997501 13.804478  2.55066748
## 5  5  NA   NA      NA     NA -0.08725202 -0.07788277  9.834865 -0.09574128
## 6  6  NA   NA      NA     NA  0.41918794  0.93787525 11.357063  0.91905737
##   is gen
## 1  0   1
## 2  0   1
## 3  0   1
## 4  0   1
## 5  0   1
## 6  0   1
```

```
ggPed  <- ggHetVar[, c("id", "dam", "sire", "is", "gen","p")]

n <- nrow(ggPed)
```

calculate A^{-1}, using the `inverseA()` function from `MCMCglmm` package:

```
ainvOut <-  inverseA(ggPed[, 1:3])
```

Store inverse of full A matrix

```
Ainv <- ainvOut$Ainv
```

Also store inbreeding values for later use

```
ggPed$f <- ainvOut$inbreeding
```

Calculation of the Q-matrix that includes genetic group proportions for each individual

This code is taken or adapted from Wolak and Reid 2017, Appendix 6.

```
naPar <- which(is.na(ggPed[, 2]))
```

Define 'focal' genetic group = "foc0" and 'immigrant' = "g1" Obtained by pasting "foc" & "g" with immigrant status "0" or "1", respectively:

```
ggPed$GG <- rep("NA", nrow(ggPed))

ggPed$GG[naPar] <- as.character(ggPed$is[naPar])
ggPed$GG[ggPed$GG == "0"] <- paste0("foc", ggPed$GG[ggPed$GG == "0"])
ggPed$GG[ggPed$GG == "1"] <- paste0("g", ggPed$GG[ggPed$GG == "1"])
```

Save original (with NA) dams and sires to not loose them

```
ggPed$damOriginal <- ggPed$dam
ggPed$sireOriginal <- ggPed$sire
ggPed[naPar, 2:3] <- ggPed[naPar, "GG"]
```

The `ggcontrib()` function propagates the genetic origin from the founders through the pedigree and results in the Q-matrix:

```
Q <- ggcontrib(ggPed[, 1:3], ggroups = c("foc0", "g1"))
```

Append the columns with genetic group contribution to the data file:

```
ggPed <- cbind(ggPed, Q)
```

Derivation of the inverse relatedness matrices A_0^{-1} and A_1^{-1} for the two genetic groups

```
ped <- pedigree(sire=ggPed[,"sireOriginal"],dam=ggPed[,"damOriginal"],label=ggPed[,"id"])
```

The components T^{-1} and D^{-1} from the Cholesky decomposition can now be directly derived from the pedigree:

```
TInv <- as(ped, "sparseMatrix")
DD <- Dmat(ped)
DInv <- Diagonal(x=1/DD)
```

We can verify that this is indeed the Cholesky decomposition by comparing the product $(T^{-1})'D^{-1}T^{-1}$ to Ainv

```
invA_test<- t(TInv) %*% DInv %*% (TInv)
sum(invA_test - Ainv)
```

```
## [1] 0
```

First, we generate A_0^{-1} for resident poppulation (group 0)

The $1/q_{ij}^2$ scaling, where q_{ij} is replaced by 10^{-6} if $q_{ij} = 0$, is given as:

```
scaling0 <- ifelse(ggPed$foc0>0,1/(ggPed$foc0)^2,1e12)
```

Then generate the $d_{ii}^{(0)}$ values:

```
DD00 <-     1- ggPed$foc0 *(1-DD)
```

And obtain $\tilde{D}_0^{-1}$ as described in equation (13) in the main text to obtain A_0^{-1}:

```
DInvTilde0 <-  Diagonal(x=1/(DD00) *scaling0)
Ainv0 <-  crossprod(sqrt(DInvTilde0) %*% TInv)
```

Note that this last operation calculates $\left(\sqrt{\tilde{D}_0^{-1}} \cdot T^{-1}\right)' \cdot \sqrt{\tilde{D}_0^{-1}} \cdot T^{-1} = (T^{-1})'\tilde{D}_0^{-1}(T^{-1})$, corresponding to equation (13) in the main text.

Repeat the same procedure for immigrants (group 1)

```
scaling1 <- ifelse(ggPed$g1>0,1/(ggPed$g1)^2,1e12)
DD11 <-     1 - ggPed$g1*(1-DD)
DInvTilde1 <-   Diagonal(x=1/(DD11)*scaling1)
Ainv1 <-  crossprod(sqrt(DInvTilde1) %*% TInv)
```

Fitting the model with INLA

Heterogeneous additive genetic variances

We need two preparations for the calculation in INLA:

1. We copy the id column multiple times for use in the `inla()` procedure:

```
ggPed$IndexA0 <- ggPed$IndexA1 <-  ggPed$id
```

This is a peculiarity of INLA, because the same id cannot be used twice in the same model, even if it encodes the same information.

2. If a animal i has a zero contribution from group j, set the respective column IDs to NA. This prevents partial breeding values a_{ij} of individuals i with $q_{ij} = 0$ from being non-identifiable.

```
ggPed$IndexA0 <- ifelse(ggPed$foc0>0, ggPed$IndexA0, NA)
ggPed$IndexA1 <- ifelse(ggPed$g1>0, ggPed$IndexA1, NA)
```

We include the inbreeding coefficient `f` and the effect `g1` that accounts for the difference in mean breeding values in the linear predictor.

In INLA, the `f()` environment encodes for random effects. The precision matrices are `Ainv0` and `Ainv1`, as derived above.

In agreement to all anlyses presented in the manuscript, penalized complexity priors PC(1,0.05) are used. The INLA formula is then given as

```
formula = p ~   f + g1 +
  f(IndexA0, model="generic0",
    Cmatrix=Ainv0,
    hyper=list(
      prec=list(initial=log(1/varf), prior="pc.prec",param=c(1,0.05))
    )
  ) +
  f(IndexA1, model="generic0",
    Cmatrix=Ainv1,
    hyper=list(
      prec=list(initial=log(1/vari),  prior="pc.prec",param=c(1,0.05))
    )
  )
```

Note that variances are encoded as log-precisions, thus initial values `log(1/varf)` and `log(1/vari)` are given to the hyperparameters. Usually we of course do not know the true variances or precisions, but users should try to initialize with a reasonable guess, and eventually verify that the results are insensitive to the initialization.

Let us now run INLA:

```
inla.model = inla(formula=formula, family="gaussian",
             data=ggPed,
             control.family=list(hyper =
                                   list( theta =
                                           list(initial=log(1/vare),  prior="pc.prec",param=c(1,0.05))
                                         )
                                 ),
             control.compute=list(dic=T)
)
```

The `control.compute=list(dic=T)` command tells INLA to calculate and store DIC, which is otherwise not done. Summaries of the posterior marginals for fixed effects and hyperparameters (i.e., for the variances) are obtained as follows, but note that the posteriors of the hyperparameters are given for the *precisions* (not for the variances):

```r
inla.model$summary.fixed
```

```
##                  mean         sd 0.025quant 0.5quant 0.975quant      mode
## (Intercept) 9.9293465 0.09448988   9.743953 9.929283  10.114914 9.9291645
## f           0.4771644 1.12319352  -1.728223 0.477114   2.680790 0.4771082
## g1          2.0703135 0.15737570   1.761311 2.070288   2.379167 2.0702498
##                      kld
## (Intercept) 0.000000e+00
## f           6.611279e-15
## g1          3.586119e-14
```

```r
inla.model$summary.hyperpar
```

```
##                                              mean         sd 0.025quant
## Precision for the Gaussian observations 0.9975748 0.07084081  0.8649242
## Precision for IndexA0                   0.5229054 0.04276554  0.4441074
## Precision for IndexA1                   0.3430099 0.03473537  0.2800205
##                                          0.5quant 0.975quant      mode
## Precision for the Gaussian observations 0.9952861  1.1437084 0.9910506
## Precision for IndexA0                   0.5210949  0.6121334 0.5174421
## Precision for IndexA1                   0.3411992  0.4164323 0.3375564
```

To obtain posteriors of the variances, we need to transform the posteriors of the precisions. That's what we need the following function for:

```r
inlaPostVariances <- function(model){

  sigma.IndexA0 <- inla.tmarginal(function(x) 1/x,
                               model$marginals.hyperpar$"Precision for IndexA0")
  e.IndexA0     <-  inla.emarginal(function(x) x, sigma.IndexA0)
  e.IndexA0_mode <-  inla.mmarginal( sigma.IndexA0)

  sigma.IndexA1 <- inla.tmarginal(function(x) 1/x,
                               model$marginals.hyperpar$"Precision for IndexA1")
  e.IndexA1     <- inla.emarginal(function(x) x, sigma.IndexA1)
  e.IndexA1_mode <- inla.mmarginal( sigma.IndexA1)

  sigma.epsilon   <- inla.tmarginal(function(x) 1/x,
                                model$marginals.hyperpar$"Precision for the Gaussian observations")
  e.Index.epsilon <- inla.emarginal(function(x) x, sigma.epsilon)
  e.Index.epsilon_mode <- inla.mmarginal(sigma.epsilon)

  results_tab <- cbind(rbind( varA0 = c(e.IndexA0,e.IndexA0_mode),
                              varA1 = c(e.IndexA1,e.IndexA1_mode),
                              varE = c(e.Index.epsilon,e.Index.epsilon_mode)),
                       1/model$summary.hyperpar[c(2,3,1),c(5,3)])
  names(results_tab) <- c("mean","mode", "2.5%", "97.5 %")
  return(results_tab)

}
```

Now get the posterior mean, mode and 95% CI:

```r
inlaPostVariances(inla.model)
```

```
##           mean      mode      2.5%   97.5 %
```

```
## varA0 1.925098 1.9073958 1.6336308 2.251708
## varA1 2.945055 2.9014502 2.4013506 3.571167
## varE  1.007463 0.9991546 0.8743487 1.156171
```

The results indicate that both posterior mode and mean are close to the values that were used to generate the data, namely $\sigma^2_{A_0} = 2$, $\sigma^2_{A_1} = 3$ and $\sigma^2_E = 1$.

Homogeneous additive genetic variances

For completeness we also carry out the analysis where we ignore group-specific additive genetic variances.

```
ggPed$IndexA <-  ggPed$id

formula = p ~   f + g1 +
  f(IndexA, model="generic0",
    Cmatrix=Ainv,
    hyper=list(
      prec=list(initial=log(1/varf), prior="pc.prec",param=c(1,0.05))
    )
  )

inla.model.homogeneous = inla(formula=formula, family="gaussian",
             data=ggPed,
             control.family=list(hyper =
                                    list( theta =
                                            list(initial=log(1/vare),  prior="pc.prec",param=c(1,0.05))
                                          )
                                  ),
             control.compute=list(dic=T)
)
```

DIC and posterior distribution of differences

Although we do not recommend to use DIC for automatic model selection, users may sometimes want to calculate it for a given model. For a fitted R-INLA object that was obtained with the `DIC=T` option above, the DIC can be obtained as follows:

```
inla.model$dic$dic
```

```
## [1] 10060.64
```

As explained in the main text, instead of using DIC we recommend to look at the posterior distribution of differences of variances instead of DIC. To obtain samples from the respective posterior, we need to activate the `inla.hyperpar.sample()` function, which draws independent samples of the joint posterior for the hyperparameters. Because INLA works with precisions τ, thus we need to transform them into variances $\sigma^2 = 1/\tau$.

```
nsamples <- 100000
sample.posterior <- inla.hyperpar.sample(n=nsamples,inla.model)

var.A0 <- 1/sample.posterior[,"Precision for IndexA0"]
var.A1 <- 1/sample.posterior[,"Precision for IndexA1"]

posterior.diff <- data.frame(var.A0 - var.A1)
```

The posterior mode, quantiles (for example 2.5% and 97.5%) and distribution can then be obtained as follows:

```r
posterior.mode(as.mcmc(posterior.diff ))
```

```
## var.A0...var.A1
##      -0.9582094
```

```r
quantile(var.A0 - var.A1,prob=c(0.025,0.975))
```

```
##       2.5%      97.5%
## -1.6724975 -0.4252626
```

```r
names(posterior.diff) <- c("diff")

library(ggplot2)
ggplot(posterior.diff,aes(x=diff)) + geom_histogram(bins=100) + theme_bw()
```

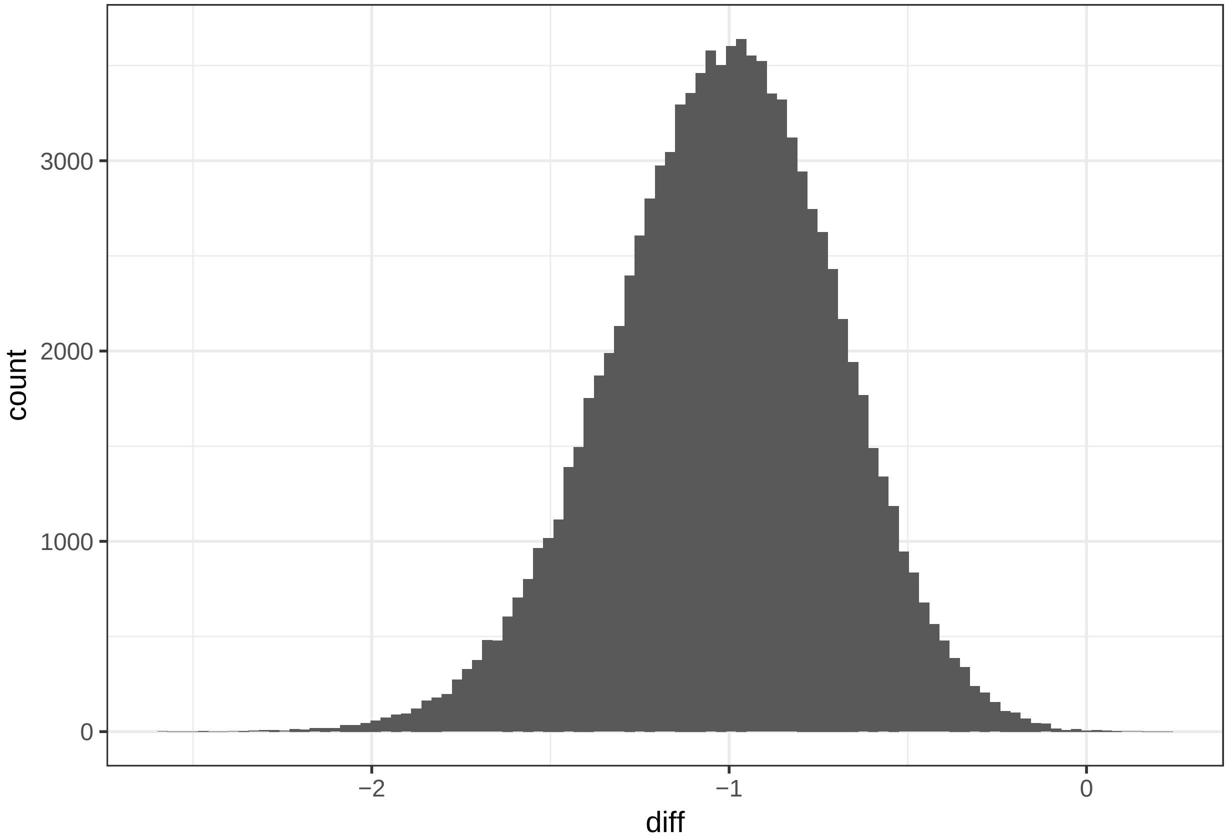

Fitting the model with MCMCglmm

Genetic group models with different additive genetic variances can also be fit using MCMC via the MCMCglmm R package (Hadfield, 2010).

Note: PC-priors are not implemented in MCMCglmm. Here we use parameter-expanded priors to ensure good mixing properties of the chains.

```r
priors <-  list(B = list(mu = rep(0, 3), V = diag(3)*1e6),
                R = list(V = diag(1), nu = 1),
                G = list(
                  G1 = list(V = diag(1), nu = 1, alpha.mu = 0, alpha.V = diag(1)*1000),
```

```
                  G2 = list(V = diag(1), nu = 1, alpha.mu = 0, alpha.V = diag(1)*1000))
                )
```

MCMCglmm requires that dimension names of the matrix have a (character) name that links columns/rows to the pedigree:

```
Ainv0 <- as(Ainv0, "dgCMatrix")
Ainv0@Dimnames[[1L]] <- as.character(ggPed$id)

Ainv1 <- as(Ainv1, "dgCMatrix")
Ainv1@Dimnames[[1L]] <- as.character(ggPed$id)
```

Let us run MCMCglmm with 11'000 iterations, a burn-in of 1'000 and a thinning of 10:

```
mcmc.model <- MCMCglmm(fixed = p ~  f + g1  ,
                       random = ~  IndexA0 + IndexA1,
                       ginverse = list(IndexA0 = Ainv0, IndexA1 = Ainv1),
                       data = ggPed,
                       prior = priors,
                       nitt = 11000, burn = 1000, thin = 10)
```

```
## Warning: 'cBind' is deprecated.
##  Since R version 3.2.0, base's cbind() should work fine with S4 objects
```

```
##
##                        MCMC iteration = 0
##
##                        MCMC iteration = 1000
##
##                        MCMC iteration = 2000
##
##                        MCMC iteration = 3000
##
##                        MCMC iteration = 4000
##
##                        MCMC iteration = 5000
##
##                        MCMC iteration = 6000
##
##                        MCMC iteration = 7000
##
##                        MCMC iteration = 8000
##
##                        MCMC iteration = 9000
##
##                        MCMC iteration = 10000
##
##                        MCMC iteration = 11000
```

```
summary(mcmc.model)
```

```
##
##  Iterations = 1001:10991
##  Thinning interval  = 10
##  Sample size  = 1000
##
##  DIC: 10051.08
```

```
##
##  G-structure:  ~IndexA0
##
##         post.mean l-95% CI u-95% CI eff.samp
## IndexA0      1.94    1.625    2.254    624.3
##
##                ~IndexA1
##
##         post.mean l-95% CI u-95% CI eff.samp
## IndexA1     3.016    2.482     3.65    857.6
##
##  R-structure:  ~units
##
##       post.mean l-95% CI u-95% CI eff.samp
## units    0.9987   0.8643    1.141    494.9
##
##  Location effects: p ~ f + g1
##
##             post.mean l-95% CI u-95% CI eff.samp  pMCMC
## (Intercept)    9.9368   9.7851  10.1293     1000 <0.001 ***
## f              0.5018  -1.7541   2.5141     1000   0.67
## g1             2.0648   1.7552   2.3685     1000 <0.001 ***
## ---
## Signif. codes:  0 '***' 0.001 '**' 0.01 '*' 0.05 '.' 0.1 ' ' 1
```

Convergence of the MCMC chain can be checked visually, and seems ok:

```r
plot(mcmc.model$Sol)
```

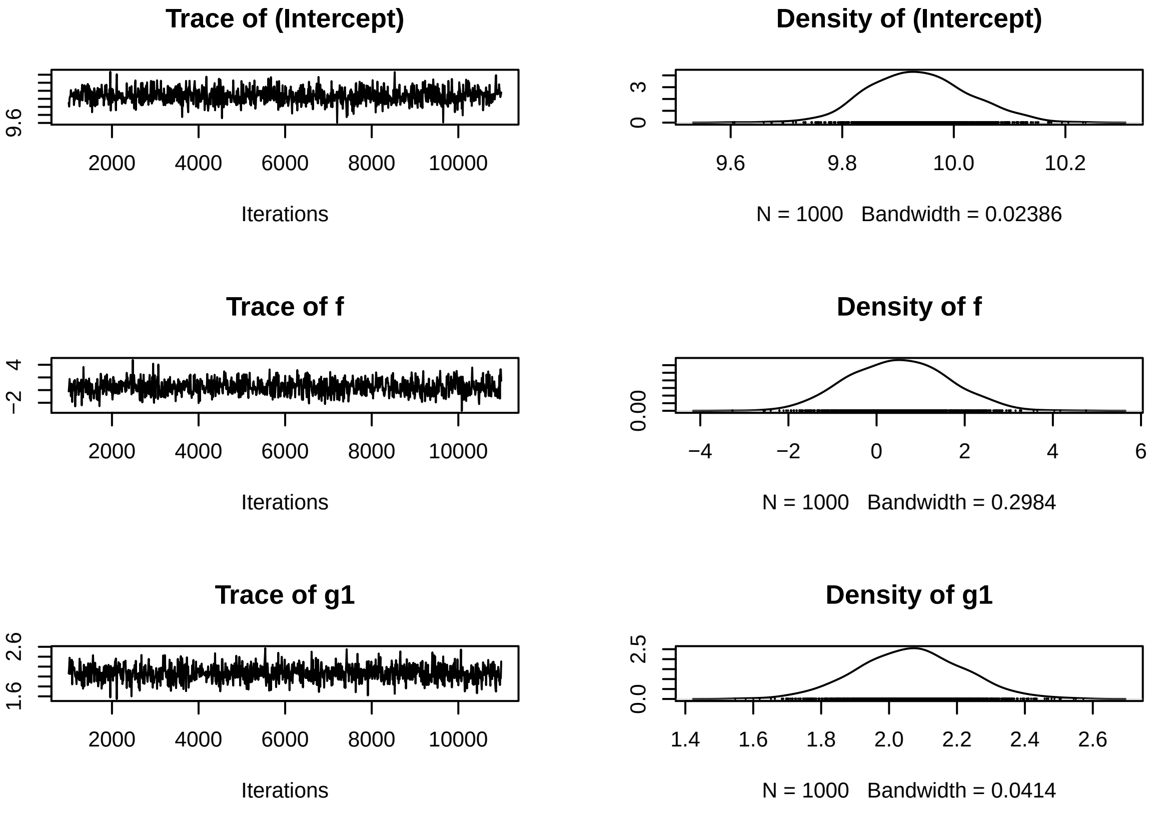

```r
plot(mcmc.model$VCV)
```

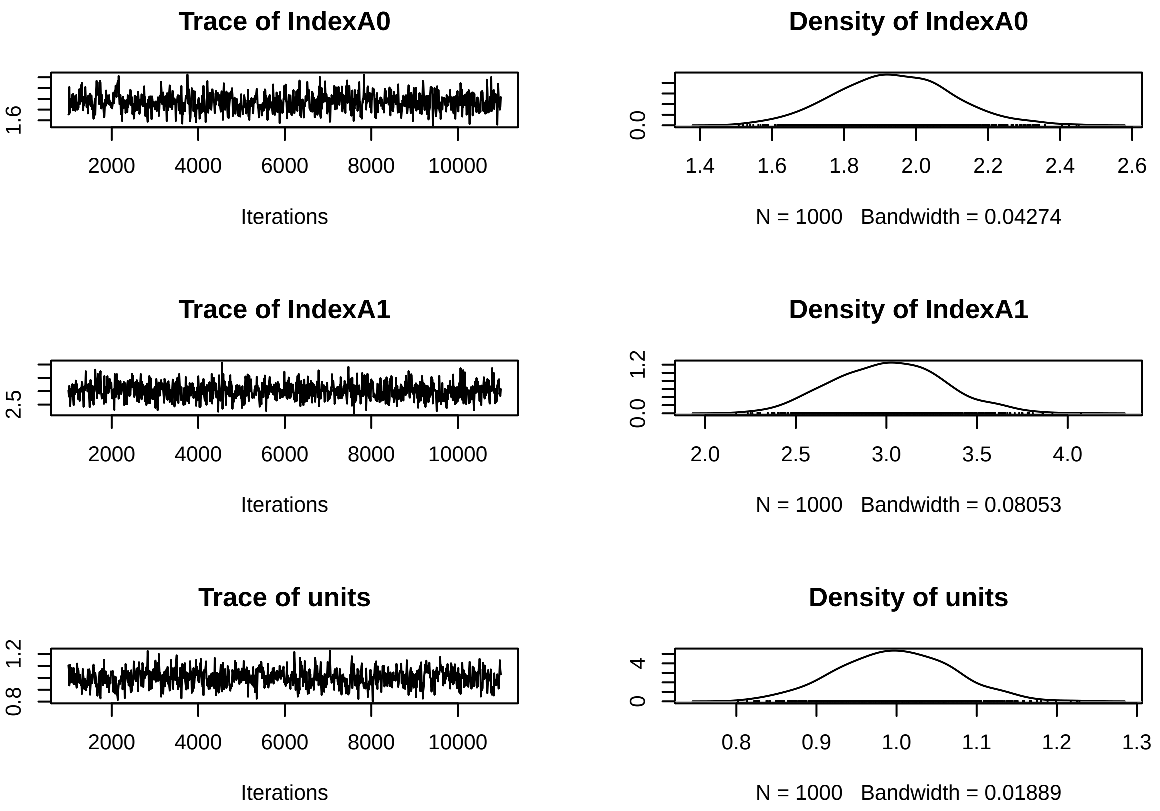

References

Hadfield, J. D. (2010). MCMC Methods for Multi-Response Generalized Linear Mixed Models: The MCMCglmm R Package. Journal of Statistical Software, 33, 1–22.

Wolak, M. and Reid, J. (2017). Accounting for genetic differences among unknown parents in microevolutionary studies: how to include genetic groups in quantitative genetic animal models. Journal of Animal Ecology 86, 7–20.
